# ﻿Integrative taxonomic study of mononchid nematodes from riparian habitats in Bulgaria. I. Genera *Mononchus* Bastian, 1865 and *Coomansus* Jairajpuri & Khan, 1977 with the description of *Mononchuspseudoaquaticus* sp. nov. and a key to the species of *Mononchus*

**DOI:** 10.3897/zookeys.1206.124237

**Published:** 2024-07-05

**Authors:** Stela Altash, Aneta Kostadinova, Vlada Peneva

**Affiliations:** 1 Institute of Biodiversity and Ecosystem Research, Bulgarian Academy of Sciences, 2 Gagarin Street, 1113 Sofia, Bulgaria Institute of Biodiversity and Ecosystem Research, Bulgarian Academy of Sciences Sofia Bulgaria

**Keywords:** Distribution, Mononchidae, morphology, phylogeny, riverine, taxonomy, 18S rDNA, 28S rDNA

## Abstract

The species diversity of the genera *Mononchus* Bastian, 1865 and *Coomansus* Jairajpuri & Khan, 1977 was assessed in a study of the mononchid nematodes from a wide range of riparian habitats in Bulgaria. Four species were identified based on morphological and morphometric data: *Coomansusparvus* (de Man, 1880), *Mononchustruncatus* Bastian, 1865, *Mononchuspseudoaquaticus***sp. nov.**, and *Mononchus* sp. The first three species were characterised both morphologically and molecularly (18S and 28S rRNA gene sequences) and the integration of these data and phylogenetic analyses provided support for their distinct species status. This paper provides detailed descriptions, morphometric data for multiple species populations, drawings and photomicrographs, and the first taxonomically verified sequences for *C.parvus* (*n* = 6), *M.truncatus* (sensu stricto) (*n* = 4) and *M.pseudoaquaticus***sp. nov.** (*n* = 3). Comparative sequence and phylogenetic analyses suggested that the utility of the 18S rRNA gene for species delimitation is rather limited at least for some species complexes within the genus *Mononchus*. At the generic and suprageneric level, the 18S and 28S rDNA phylogenies both recovered the three genera represented by two or more species (*Mononchus*, *Mylonchulus*, and *Parkellus*) as monophyletic with strong support, the Mononchidae as paraphyletic, the Anatonchidae as monophyletic, and there was no support for a sister-group relationship between *Mylonchulus* and *Mononchus*. A key to the species of *Mononchus* is provided to facilitate the identification of the currently recognised 31 species.

## ﻿Introduction

Riparian zones, i.e., the ecotones between aquatic and terrestrial ecosystems, represent areas of high biodiversity caused by the diversity of habitats and heterogeneous environmental conditions they provide. Both plant and animal diversity are high in these areas with impressive levels of faunal diversity in riparian soils (Décamps et al. 2009). Soils in these functionally unique ecosystems are also important for sustaining diverse nematode communities, e.g., Décamps et al. (2009) estimated the number of species of nematodes in riparian soils to be greater than 5000. Because soil nematodes are abundant and functionally diverse, they can serve as useful indicators of food-web structure and complexity (Bongers and Ferris 1999; Ferris et al. 2001; Neher 2001; Ferris 2005); nematode communities are also important for ecosystem functions. These features justify the increased interest in studying free-living nematode communities and the ecosystem functions they perform in both undisturbed and disturbed riparian zones and riparian corridors (e.g., Young-Mathews et al. 2010; Briar et al. 2012; Hodson et al. 2014). Notably, nematodes are usually identified to the genus/family level in these ecological studies due to difficulties in the identification based on morphological characters and/or the lack of taxonomic expertise, so there is a lack of species-level assessments on larger-scale processes in riparian zones (Bardgett et al. 2001).

Sequence-based tools such as barcoding have proven successful in accelerating identification of previously characterised species or in detecting cryptic species (Palomares-Rius et al. 2014, 2022; Archidona-Yuste et al. 2023). Currently, two nuclear loci are considered to be most relevant to barcoding of nematodes, the small subunit ribosomal RNA gene (SSU or 18S) and the large subunit ribosomal RNA gene (LSU or 28S), the first being the best sampled gene in nematodes, and the second being the subject of increased interest. However, key for the successful application of barcoding for nematodes is the availability of a database of taxonomically verified sequences, i.e., associated with species identification based on detailed morphological characterisation and morphological vouchering (physical and “virtual” vouchers sensu De Ley et al. 2005). Although the number of species of the order Mononchida Jairajpuri, 1969 with sequences available for both nuclear loci indicated above is limited, a recent trend towards building a combined evidence database is promising (Kim et al. 2018; Tabolin and Kolganova 2020; van Rensburg et al. 2021; Vu 2021; Vu et al. 2021a, 2021b; Shokoohi and Moyo 2022).

In a study of the free-living nematodes from a wide range of riparian habitats in Bulgaria, we have collected several species of three families of the order Mononchida. These have been characterised both morphologically and molecularly. This paper presents the results of the integrative taxonomic study of the species of *Coomansus* Jairajpuri & Khan, 1977 and *Mononchus* Bastian, 1865 (family Mononchidae Chitwood, 1937), and phylogenetic analyses that delineate the species and establish their relationships within the suborder Mononchina Kirjanova & Krall, 1969 based on partial sequences of the 28S and 18S rRNA genes.

Species of the order Mononchida occur in both aquatic and terrestrial habitats. Species of the genus *Mononchus* are aquatic nematodes, occasionally occurring in wet terrestrial habitats (Zullini and Peneva 2006; Andrássy 2009) unlike the species of the second genus considered here, *Coomansus*, which predominantly dwell in terrestrial habitats. Currently, the genus *Mononchus* contains 30 species (Andrássy 2011a; Shah and Hussain 2016; Gagarin and Naumova 2017; Ishaque et al. 2022). According to Andrássy (2009) the number of valid species of *Coomansus* is 28. Subsequently, five new species have been described (Andrássy 2011b; Shah and Hussain 2015; Vu 2021). Ahmad and Jairajpuri (2010) transferred the species of the *Coomansus* “*zschokkei*-group” to the genus *Parkellus* Jairajpuri, Tahseen & Choi, 2001; however, the validity of *Parkellus* is not widely accepted (e.g., Zullini and Peneva 2006; Andrássy 2009, 2011b).

In Bulgaria, two species of the genus *Coomansus*, *C.parvus* (de Man, 1880) Jairajpuri & Khan, 1977 and *Coomansuszschokkei* (Menzel, 1913) Jairajpuri & Khan, 1977 have been reported (Iliev and Ilieva 2016). Two further species of the genus *Mononchus*, *M.truncatus* Bastian, 1865 and *M.aquaticus* Coetzee, 1968 have also been recorded; however, morphological data have not been provided (Andrássy 1958; Katalan-Gateva 1962, 1965; Stoichev 1996; Lazarova et al. 2004; Stoichev and Chernev 2011; Stoichev and Varadinova 2011). Only the latter two species have been reported in aquatic habitats.

## ﻿Materials and methods

### ﻿Sampling, nematode isolation, and processing

More than 150 soil and litter samples were collected at 76 localities in different riparian zones in Bulgaria. Multiple core soil samples (3 per site) were collected at a depth of 40–60 cm from each habitat (sampling site of 15 × 15 m or along the riverbank) around the roots of the dominant tree species; litter samples were collected simultaneously.

Nematodes were extracted from soil (at least 400 g) and litter (at least 20 g) samples using a decanting and sieving technique and a modified Baerman funnel method with 48 h of exposition and counted alive. Thereafter, the nematodes were gently heated at 63 °C for 2 min and fixed in 4% formaldehyde, 1% glycerine, dehydrated, and mounted on permanent slides in anhydrous glycerine with paraffin as a support for the cover slide (Seinhorst 1959). Morphological examination was carried out and measurement taken under a light microscope (Olympus BX41, Tokyo, Japan) equipped with a digitising tablet (CalComp Drawing Board III) and using the DIGITRAK 1.0 f program (Philip Smith, the John Hutton Institute, Dundee, UK). Drawings were prepared using an Olympus BX51 compound microscope with differential interference contrast (DIC). Photomicrographs were taken with Axio Imager.M2 microscope (Carl Zeiss, Oberkochen, Germany) equipped with a digital camera (ProgRes C7) and CapturePro 2.8 software (Jenoptic).

All measurements in the descriptions and tables are in micrometres unless stated otherwise and are given as the mean ± standard deviation followed by the range in parentheses. A standard set of De Man indices was calculated for each specimen as follows: *L*, body length; *V*, distance from vulva to anterior end of body as % of body length; *a*, body length/greatest body diameter; *b*, body length/distance from anterior end to pharyngo-intestinal valve; *c*, body length/tail length; *c*’ tail length/tail diameter at anus; *G1* anterior female gonad length as % of body length; *G2* posterior female gonad length as % of body length (De Man 1876, 1880).

### ﻿DNA isolation, amplification, and sequencing

Specimens intended for the molecular study were identified on temporary mounts; a standard set of photomicrographs was taken for each specimen. Genomic DNA (gDNA) was isolated using 5% suspension of deionised water and Chelex®, containing 0.1 mg/ml proteinase K; samples were incubated at 56 °C for 3 h or overnight, boiled at 90 °C for 8 min, and centrifuged at 14,000× g for 10 min. Two genetic markers were sequenced, the small (18S) and the large (28S) ribosomal subunit RNA coding regions.

Partial fragments of the 28S rRNA gene (domains D1-D3; ~ 1000 bp) were amplified using the forward primer LSU5 (5’-TAG GTC GAC CCG CTG AAY TTA AGC A-3’) (Littlewood et al. 2000) and the reverse primer 1500R (5’-GCT ATC CTG AGG GAA ACT TCG-3’) (Tkach et al. 1999). Nearly complete (~ 1600–1700 bp) fragments of the 18S rRNA gene were amplified in two partially overlapping fragments using the primer sets 988F (forward: 5’-CTC AAA GAT TAA GCC ATG C-3’) and 1912R (reverse: 5’-TTT ACG GTC AGA ACT AGG G-3’) for the first fragment, and 1813F (forward: 5’-CTG CGT GAG AGG TGA AAT-3’) and 2646R (reverse: 5’-GCT ACC TTG TTA CGA CTT TT-3’) for the second fragment (Holterman et al. 2006).

PCR amplifications were performed in a total volume of 25 µl using Illustra ™ PuReTaq™ Ready-To-Go™ PCR beads (GE Healthcare, Chicago, USA; Cat. # 27-9559-01). In the case of poor amplification, the PCR reactions were performed with 2× MyFi™ DNA Polymerase mix (Bioline Inc., Taunton, USA; Cat. # BIO-25049) in a total volume of 20 μl, containing 8 pmol of each primer and ~ 50 ng of gDNA. The amplification profile for 28S rDNA comprised an initial denaturation at 94 °C for 5 min (or 3 min when using MyFi™ DNA Polymerase mix) followed by 40 cycles (30 s at 94 °C; 30 s at 55 °C; and 2 min at 72 °C), and a final extension step at 72 °C for 7 min. The following amplification profile was used for 18S rDNA: initial denaturation at 94 °C for 5 min, followed by 5 cycles (30 s at 94 °C; 30 s at 45 °C; 70 s at 72 °C) and 35 cycles (30 s at 94 °C; 30 s at 54 °C; 70 s at 72 °C), and a final extension step at 72 °C for 5 min. PCR amplicons were purified and sequenced directly for both strands using the PCR primers (and in some cases the internal primers 300F, ECD2 and LSU1200R (Littlewood et al. 2000) for 28S rDNA) at Macrogen Europe (Amsterdam, the Netherlands). Contiguous sequences were assembled, quality checked, and edited manually using MEGA7 (Kumar et al. 2016) and subjected to a BLASTn search on the NCBI GenBank database.

### ﻿Phylogenetic analyses

The newly generated 18S rDNA and 28S rDNA sequences were aligned separately using MUSCLE implemented in MEGA7 (Kumar et al. 2016) with representative sequences available in the GenBank database. First, an exploratory neighbour-joining (NJ) analysis was carried out on an untrimmed 28S rDNA alignment (domains D1-D3), including representative sequences for *Mononchus* spp. and *Coomansus* spp. to assess the associations of the newly generated sequences from riparian nematode populations sampled in Bulgaria.

Secondly, two alignments were constructed comprising sequences for species of three families of the suborder Mononchina: Anatonchidae Jairajpuri, 1969, Mononchidae, and Mylonchulidae Jairajpuri, 1969. These alignments were trimmed to the length of the shortest sequence. The 28S rDNA alignment (domains D2-D3) contained 33 sequences for representatives of ten genera of the three families and the 18S rDNA alignment contained 32 sequences for representatives of ten genera of the three families.

Phylogenetic relationships were estimated by conducting maximum likelihood (ML) analyses as implemented in MEGA7. Prior to analyses, the best-fitting models of nucleotide substitution were estimated based on the Akaike information criterion (AIC); these were the Tamura 3-parameter model (T92) including estimates of invariant sites and among-site rate heterogeneity (T92+I+G) for the 18S rDNA alignment and the Kimura 2-parameter model (K2) with among-site rate heterogeneity (K2+G) for the 28S rDNA alignment. Nodal support was estimated by performing 1000 bootstrap pseudoreplicates. *Mermisnigrescens* Dujardin, 1842 was used as the outgroup in the analyses of both alignments based on the phylogeny published by Holterman et al. (2006). Genetic distances (number of nucleotide positions and uncorrected p-distance) were calculated in MEGA7.

## ﻿Results

### ﻿Overview of the morphological identification and the novel molecular and distributional data

A total of 17 populations of *Coomansus* spp. and *Mononchus* spp. were collected in soil and litter samples from habitats with various vegetation types along 12 rivers (Arda, Danube, Devinska, Dyavolska, Grafska, Lopushnitsa, Maritsa, Rezovska, Shirokoleshka, Trigradska, Vedena, and Veleka) in eight provinces in Bulgaria (Burgas, Kardzhali, Lovech, Montana, Plovdiv, Silistra, Smolyan, and Sofia). In each locality, the nematode populations were recovered around the roots of the dominant tree species (predominantly *Salix* spp., but also *Alnusglutinosa* (L.), *Carpinusbetulus* L., *Fagussylvatica* L., *Fraxinusexcelsior* L., *Populus* sp., *Ulmuslaevis* Pall., and *Ulmus* sp.) (Table 1).

**Table 1.** Summary data for the populations of *Coomansusparvus* and *Mononchus* spp. studied in 17 riparian habitats in Bulgaria.

Species	River	Locality	Coordinates	Elevation (m)^a^	Associated tree species (habitat)	Date (Collector)
*Coomansusparvus* (de Man, 1880)	Lopushnitsa (Balkan Mountains)	Near Kaleytsa, Lovech Province	42°55'34"N, 24°38'38"E	~ 440	*Acer* sp. (litter)	9.05.2021 (VP)
	Arda (Rhodope Mountains)	Dyavolski Most, Kardzhali Province	41°37'14"N, 25°06'53"E	~ 460	*Ulmus* sp. (soil)	29.08.2020 (VP)
			41°37'22"N, 25°06'54"E	~ 440	*Populus* sp. (soil)	
	Vedena (Vitosha Mountain)	Near Zheleznitsa, Sofia Province	42°32'05"N, 23°20'57"E	~ 1200	*Fagussylvatica* L. (litter)	4.04.2022 (SA, VP)
	Devinska (Rhodope Mountains)	Near Devin, Smolyan Province	41°45'21"N, 24°20'02"E	~ 880	*Carpinusbetulus* L. (soil)	20.05.2019 (SA)
*Mononchustruncatus* Bastian, 1865	Shirokoleshka (Rhodope Mountains)	Shiroka Laka, Smolyan Province	41°40'26"N, 24°35'51"E	~ 1120	*Salix* sp. (soil)	23.05.2019 (SA)
	Maritsa (Upper Thracian Plain)	Near Plovdiv, Plovdiv Province	42°09'N, 25°50'E	~ 153	*Salix* sp. (soil)	18.10.1995 (VP)
	Trigradska (Rhodope Mountains)	Teshel, Smolyan Province	41°40'18"N, 24°21'13"E	~ 860	*Salix* sp. (litter)	23.05.2019 (SA)
	Dyavolska (Strandzha Mountains)	Near Primorsko, Burgas Province	42°15'34"N, 27°44'18"E	~ 10	*Fraxinusexcelsior* L. (soil)	6.06.2019 (SA)
	Rezovska (Strandzha Mountains)	Slivarovo, Burgas Province	41°57'N, 27°40'E	~ 240	*Ulmuslaevis* Pall. (soil)	22.10.2008 (RS)
	Danube (Southern Dobruja)	Vetren, Silistra Province	44°08'24"N, 27°01'47"E	~ 20	*Salix* sp. (soil)	5.07.2021 (VP)
	Veleka	Brodilovo, Burgas Province	42°04'53"N, 27°51'33"E	~ 15	*Alnusglutinosa* (L.) (soil)	4.06.2019 (SA)
*Mononchuspseudoaquaticus* sp. nov.	Shirokoleshka (Rhodope Mountains)	Shiroka Laka, Smolyan Province	41°40'26"N, 24°35'51"E	~ 1120	*Salix* sp. (soil)	23.05.2019 (SA)
	Maritsa (Upper Thracian Plain)	Near Plovdiv, Plovdiv Province	42°09'N, 25°50'E	~ 153	*Salix* sp. (soil)	18.10.1995 (VP)
	Veleka	Brodilovo, Burgas Province	42°04'53"N, 27°51'33"E	~ 15	*Alnusglutinosa* (L.) (soil)	4.06.2019 (SA)
	Danube (Southern Dobruja)	Vetren, Silistra Province	44°08'24"N, 27°01'47"E	~ 20	*Salix* sp. (soil)^b^	5.07.2021 (VP)
	Danube (Southern Dobruja)	Komluka Island, Silistra Province	44°08'03"N, 27°03'40"E	~ 20	*Populus* sp. (soil)	5.07.2021 (VP)
*Mononchus* sp.	Grafska, inflow of River Kopilovtsi (Balkan Mountains)	Waterfall “Durshin skok”, near Kopilovtsi, Montana Province	43°19'40"N, 22°51'01"E	~ 1048	*Fagussylvatica* L. (soil)	27.07.2000 (VP)

Abbreviations: RS, Rabia Soufi; SD, Stela Altash; VP, Vlada Peneva. ^a^ Metres above sea level. ^b^ Type-population.

Four species were identified based on morphological data: *C.parvus* (4 populations), *M.truncatus* (7 populations), *Mononchuspseudoaquaticus* sp. nov. (5 populations), and *Mononchus* sp. (1 population). The geographical distribution of *Mononchus* spp. (9 localities) did not overlap that of the single species of *Coomansus* recovered during the study (4 localities) (Table 1). Of note, populations of the two widespread species of *Mononchus*, *M.truncatus* and *M.pseudoaquaticus* sp. nov., co-occurred in four localities (along riverbanks of the rivers Danube, Maritsa, Shirokoleshka, and Veleka).

Although an attempt was made to obtain representative 28S rDNA sequences for all species populations, the success rate was generally low. A total of nine sequences were generated, four for *C.parvus* (1017–1037 bp), three for *M.truncatus* (987–1041 bp), and two for *M.pseudoaquaticus* sp. nov. (910–1042 bp). Four of the sequenced populations were selected for generating representative 18S rDNA sequences (1630–1682 bp; 2 for *C parvus*, 1 for *M.truncatus*, and 1 for *M.pseudoaquaticus* sp. nov.). No sequences were generated for *Mononchus* sp. The newly generated 28S rDNA sequences showed very low intraspecific genetic divergence (0–2 nt positions, i.e., 0.2% for sequences for *C.parvus* and *M.truncatus*, and identical sequences for *M.pseudoaquaticus* sp. nov.); the two 18S rDNA sequences for *C.parvus* were also identical.

### ﻿Taxonomy

#### ﻿Genus *Coomansus* Jairajpuri & Khan, 1977

##### ﻿ Coomansus parvus

Taxon classificationAnimaliaMononchidaMononchidae

(de Man, 1880) Jairajpuri & Khan, 1977

6E202E8A-3A43-548E-8B1C-40D2D711212E

Figs 1 , 2

###### Description.

**Female** [Based on 10 specimens from 3 localities; see Table 2 for measurements]. Body short, 0.70–1.15 mm, J- or C-shaped upon relaxation, body diameter at mid-body 47–53. Cuticle smooth under light microscope (very faint striation observed in one specimen, Fig. 2H), 2–3 thick along most of body, 3–4 thick in post-anal region. Lip region offset, cephalic and labial papillae prominent, conical, of almost same size. Amphid apertures oval, 5 ± 0.4 (4–5) (*n* = 6) wide, situated anterior to dorsal tooth apex, at 10–14 from anterior end. Buccal capsule oval, somewhat flattened at base, 1.6–1.9 as long as wide or 0.9–1.2 times as long as lip region width; its ventral wall 1.5–2.5 thick, dorsal wall posterior to dorsal tooth ~ 3 thick. Dorsal tooth small, its anterior margin 3.0 ± 0.5 (2–4) wide, located near middle of buccal capsule, tooth apex at 9 ± 1 (8–11) from anterior end of buccal capsule. Nerve-ring at 97 ± 6 (90–103) (*n* = 6) from anterior end of body. Excretory pore posterior to nerve-ring, small, well visible. Reproductive system amphidelphic. Genital branches almost symmetrical; anterior branch 134 ± 50 (80–253) (*n* = 9) long; posterior branch 115 ± 19 (80–133) (*n* = 9) long. Ovaries well developed; anterior ovary 60–105 (*n* = 7) long; posterior ovary 70–140 (*n* = 7) long. Oviduct with marked *pars dilatata oviductus*, ~ 30 wide. Uteri very short. Two uterine eggs present in one female measuring 81 × 40 and 90 × 38. Vagina with straight walls, its length representing 25–33% of corresponding body width; *pars refringens vaginae* as 2 oval to drop-shaped smooth sclerotised pieces, 3–4 long and 2–3 wide; *pars distalis vaginae* ~ 3 long. Vulva a transverse slit; *pars refringens vaginae* protruding in some specimens (Fig. 2I). Rectum 0.7–0.8 times as long as body diameter at anus. Tail conoid, ventrally arcuate, with finely rounded tip. Caudal glands and spinneret absent. Caudal pores two pairs. **Male**: Not found.

**Figure 1.**
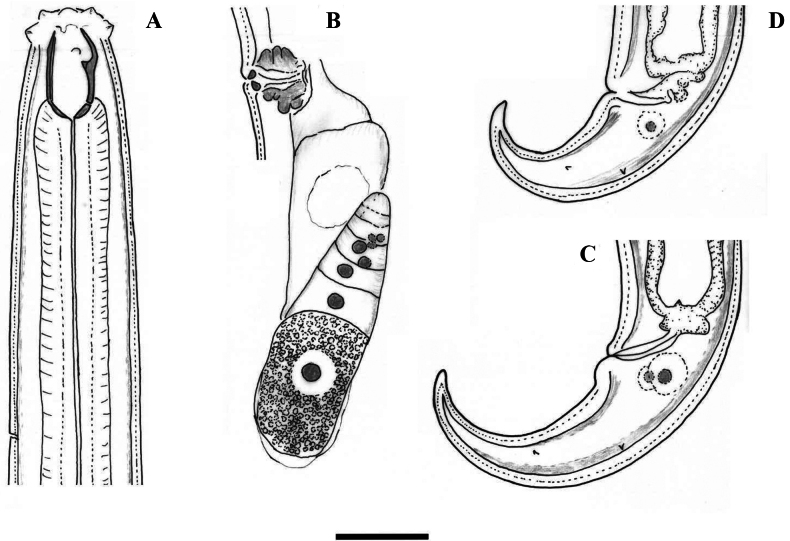
Line drawings of *Coomansusparvus* (de Man, 1880) Jairajpuri & Khan, 1977. Female specimens from populations collected from riverbanks of the rivers Vedena (**A**, **B**, **D**) and Arda (**C**): **A** anterior region **B** posterior genital branch **C, D** tail end. Scale bar: 25 µm.

**Figure 2.**
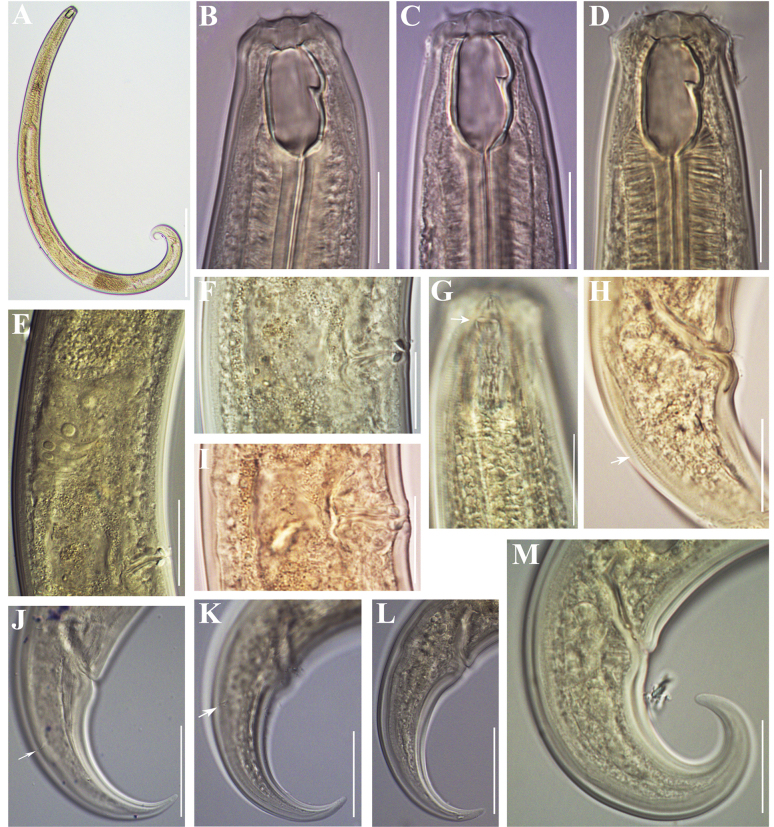
Photomicrographs of *Coomansusparvus* (de Man, 1880) Jairajpuri & Khan, 1977. Female specimens from populations collected from riverbanks of the rivers Lopushnitsa (**A, B, E–G, M**), Vedena (**C, D, H, I, K, L**), and Arda (**J**): **A** entire body **B–D, G** anterior region (amphid opening arrowed in **G**) **E**, **F**, **I** reproductive system (**E** anterior genital branch **F, I** vulval region showing *pars refringens vaginae*) **H, J–M** tail (cuticle striation arrowed in **H**; caudal pores arrowed in **J** and **K**). Scale bars: 200 µm (**A**); 20 µm (**B–D, F–I, M**); 50 µm (**E, J–L**).

**Table 2.** Morphometric data for females of *Coomansusparvus* collected in four riparian localities in Bulgaria.

Locality	Near Kaleytsa, Lovech Province	Dyavolski Most, Kardzhali Province		Near Zheleznitsa, Sofia Province
River	Lopushnitsa (Balkan Mountains)	Arda (Rhodope Mountains)		Vedena (Vitosha Mountain)
Habitat	*Acer* sp. (litter)	*Ulmus* sp. (soil)	*Populus* sp. (soil)	*Fagussylvatica* (litter)
*n*	(*n* = 1)	(*n* = 1)	(*n* = 2)	(*n* = 6)
*L* (mm)	1.05	0.70	0.90, 1.07	0.96 ± 0.14 (0.83–1.15)
*a*	18.7	12.9	17, 17	19.0 ± 2.5 (16.2–21.7)
*b*	3.6	3.7	3.5, 3.5	3.2, 3.4, 3.5 (*n* = 3)
*c*	14.8	11.7	12.2, 12.9	12.7 ± 1.1 (11.5–14.0)
*c*’	2.3	2.1	2.4, 2.3	2.3 ± 0.3 (2.0–2.7)
*V* (%)	62.0	59.9	61.3, 62.2	62.5 ± 1.6 (59.6–64.5)
*G1* (%)	9.2	11.4	12.2, 10.5	13.8 ± 2.4 (12.0–17.3) (*n* = 5)
*G2* (%)	12.6	11.3	10.2, 11.4	12.6 ± 1.5 (11.6–15.1) (*n* = 5)
Buccal capsule length	24	22	23, 26	26 ± 1 (25–27)
Buccal capsule width	14	14	14, 15	15 ± 0.4 (14–15)
Tooth apex from anterior end of buccal capsule	9	11	8, 8	9 ± 0.4 (9–10)
Position of tooth apex (%)^a^	38	–	36, 32	35 ± 2 (33–38)
Excretory pore from anterior end	116	–	110, 113	117 ± 15 (97–131)
Nerve-ring from anterior end	92	–	90, –	100 ± 4 (94–103) (*n* = 4)
Pharynx length	294	189	258, 302	303, 324, 324 (*n* = 3)
Lip region height	8	7	8, 8	8 ± 1 (7–10) (*n* = 4)
Lip region width	25	23	25, 25	24 ± 3 (22–28) (*n* = 4)
Amphid from anterior end	11	14	14, 11	10 (*n* = 2)
Maximum body diameter	56	54	53, –	50 ± 2 (48–53)
Body diameter at pharynx base	49	49	51, –	46 ± 3 (43–50) (*n* = 5)
Body diameter at mid-body	49	53	51, –	50 ± 2 (48–53)
Body diameter at vagina	56	54	53, –	50 ± 2 (48–53)
Body diameter at anus	31	28	31, 36	33 ± 2 (31–36)
Anterior genital branch length	96	80	110, 112	161 ± 53 (118–253) (*n* = 5)
Posterior genital branch length	132	80	92, 122	122 ± 11 (106–133) (*n* = 5)
Anterior ovary length	90	70	60, 80	100, 105, 105
Posterior ovary length	105	75	70, 100	86, 110, 140
Vagina length	17	17	16, 18	14 ± 2 (12–16) (*n* = 5)
Rectum length	23	21	24; 26	23 ± 2 (21–25)
Tail length	71	60	74; 83	75 ± 8 (66–85)

^a^ Distance from tooth apex to anterior end of buccal capsule as % of buccal capsule length from its anterior

###### Voucher material.

Ten specimens are deposited in the Nematological Collection of the Institute of Biodiversity and Ecosystem Research, Bulgarian Academy of Sciences, Bulgaria, under the accession numbers IBER-BAS NC 49/1, IBER-BAS NC 51/2, IBER-BAS PN 68/4 litter, IBER-BAS NC 88/4, IBER-BAS NC 88/7-9. Photovouchers for the sequenced specimens are provided in Suppl. material 1: figs S1–S3.

###### Habitats and localities.

Soil around *Ulmus* sp., *Populus* sp., and *C.betulus* and litter around *F.sylvatica* and *Acer* sp. along riverbanks of the rivers Arda, Lopushnitsa, Vedena, and Devinska (see Table 1 for details).

###### Representative DNA sequences.

28S rRNA gene (GenBank: PP768895–PP768898); 18S rRNA gene (GenBank: PP768899 and PP768900).

###### Distribution.

Almost cosmopolitan, except in Australia (Andrássy 2009). In Bulgaria, *C.parvus* has been reported with no morphological evidence supporting identification in soil samples from an oak forest in Burgas Province (Aleksiev et al. 1998), from beech forests in Strandzha Mountain (Strandzha Nature Park, protected zones “Bjalata prust” and “Propada”; Iliev and Ilieva 2014), and from arable lands in Sofia (Katalan-Gateva 1968) and Kazanlak provinces (Katalan-Gateva et al. 1981). Iliev and Ilieva (2016) described and illustrated *C.parvus* based on a large population of females in soil samples from one habitat in the Rhodope Mountains. The present study provides the second documented record of *C.parvus* in Bulgaria, the first record of this species in litter samples, and four new localities in three provinces (Tables 1, 2).

###### Remarks.

Morphologically, the present material belongs to and was identified as *C.parvus*. Some variation was detected in the present material with single specimens from three populations sampled in the Rhodope and Balkan Mountains showing lower values for *L*, *a*, *G1*, *G2*, the length of the genital branches, ovaries, and tail, and greater values for the distance of the amphid from anterior end compared with the population from Vitosha Mountain (Table 2). We consider these small metrical differences to represent intraspecific variation; this was confirmed by the very low levels of genetic divergence (see above).

The morphometric data for the present material fall within the range given by Andrássy (2011b), except for the slightly greater values for the width of the buccal capsule (14–15 vs 10–12 µm). Comparisons with published descriptions of *C.parvus* revealed an overlap with the morphometric data of the present material but also a greater variation with higher upper limits of variation for the published ranges of body length (Zullini et al. 2002; Ahmad and Jairajpuri 2010; Ishaque et al. 2022) and most of the indices, and lower ranges for the width of the buccal capsule in four populations falling below the range recorded in the present specimens (Suppl. material 2: table S1). It is worth noting that there was an overall good agreement with the descriptions and morphometric data for a population of *C.parvus* collected in Bulgaria by Iliev and Ilieva (2016) and especially with a population used for generating 18S rDNA and 28S rDNA sequences for this species described by Tabolin and Kolganova (2020).

However, the material described by Ishaque et al. (2022) showed little overlap with the published descriptions and the present material, with ranges for a number of characters falling outside the known ranges for *C.parvus*: outside the upper limits of variation (*L*, *V*, buccal capsule length and width, lip region width, and rectum length); and outside the lower limits of variation (*G1*, *G2*, and position of tooth apex) (Suppl. material 2: table S1). This material keys down to *C.indicus* Jairajpuri & Khan, 1982 in the key to species of *Coomansus* by Andrássy (1993, 2009) and to *C.ulsani* Choi, Khan & Lee, 1999 in the key by Vu (2021) but does not agree completely with the data for these species. Clearly, the material of Ishaque et al. (2022) does not belong to *C.parvus* but definite identification is not possible based on the available data and illustrations (also see comments in Suppl. material 2: table S1).

#### ﻿Genus *Mononchus* Bastian, 1865

##### ﻿ Mononchus pseudoaquaticus sp. nov.

Taxon classificationAnimaliaMononchidaMononchidae

F3B1A966-8899-57FD-8C7F-BAD785ABB14B

https://zoobank.org/DBD4723B-BBB9-4F7D-BB79-4DDE8CECEC16

**Figure 3.**
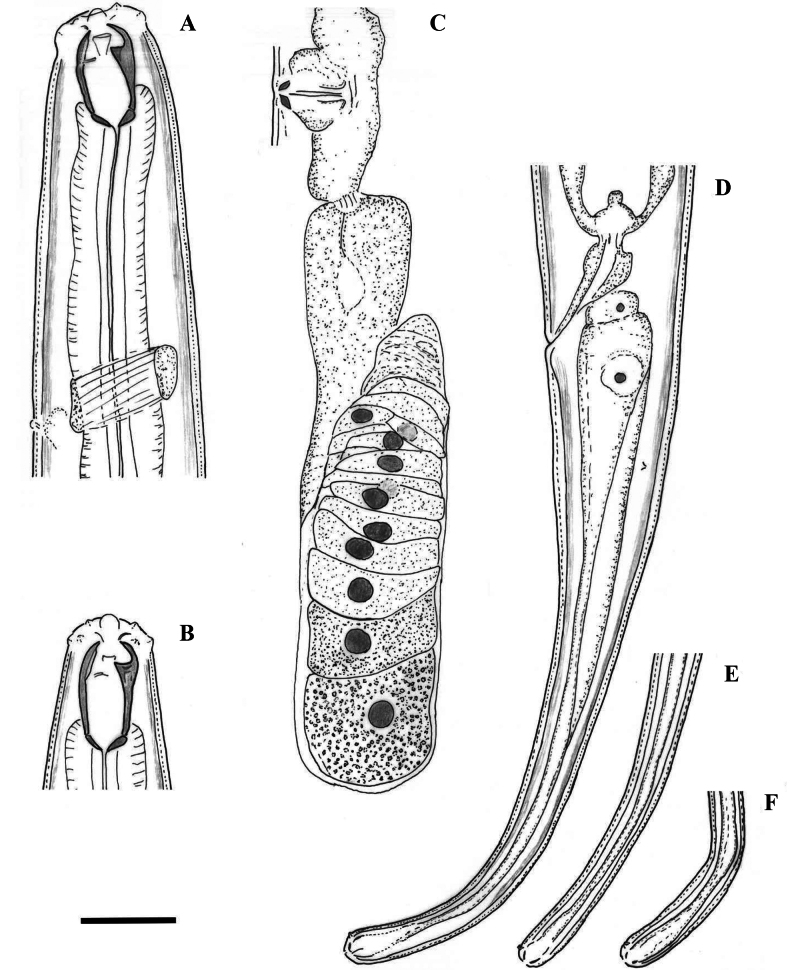
Line drawings of *Mononchuspseudoaquaticus* sp. nov. Holotype female (**A, C, D**) and paratype specimens (**B, E, F**): **A, B** anterior region **C** posterior genital branch **D** tail region **E, F** tail tip. Scale bar: 25 µm.

**Figure 4.**
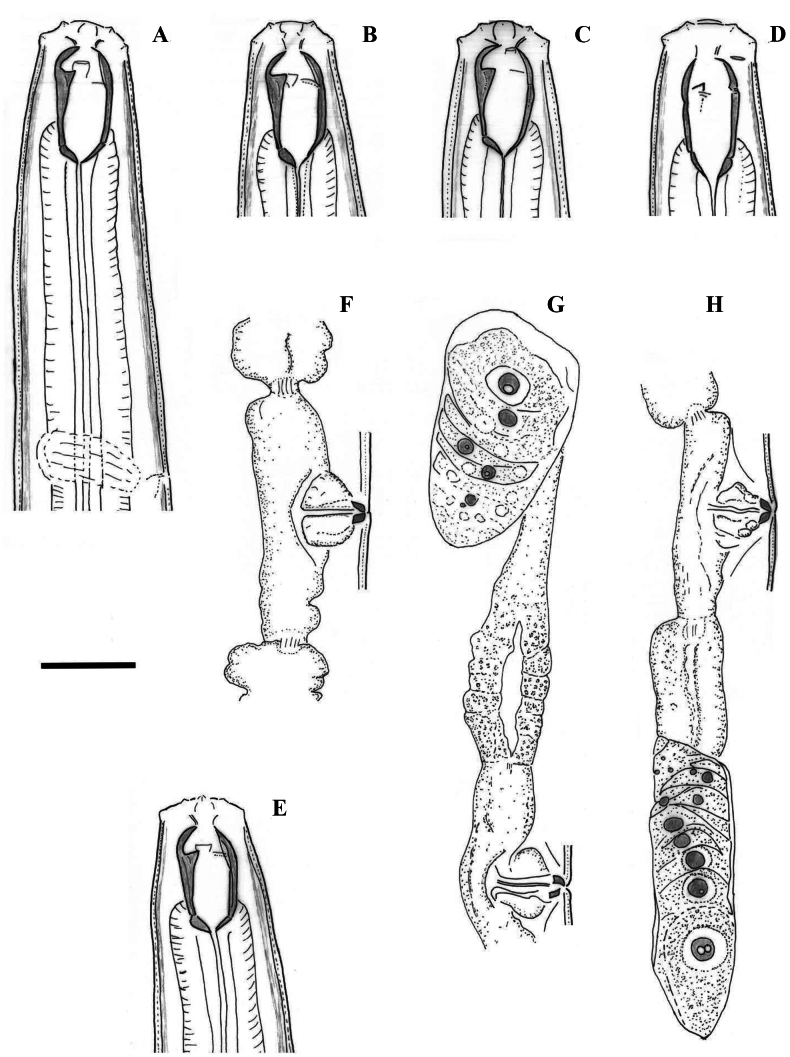
Line drawings of *Mononchuspseudoaquaticus* sp. nov. Paratype females from populations collected from riverbanks of the rivers Shirokoleshka (**A, H**), Maritsa (**B, F**), Veleka (**E, G**) and Danube (**C, D**): **A–E** anterior region **F** vulval region **G** anterior genital branch **H** vulval region and posterior genital branch. Scale bar: 25 µm.

**Figure 5.**
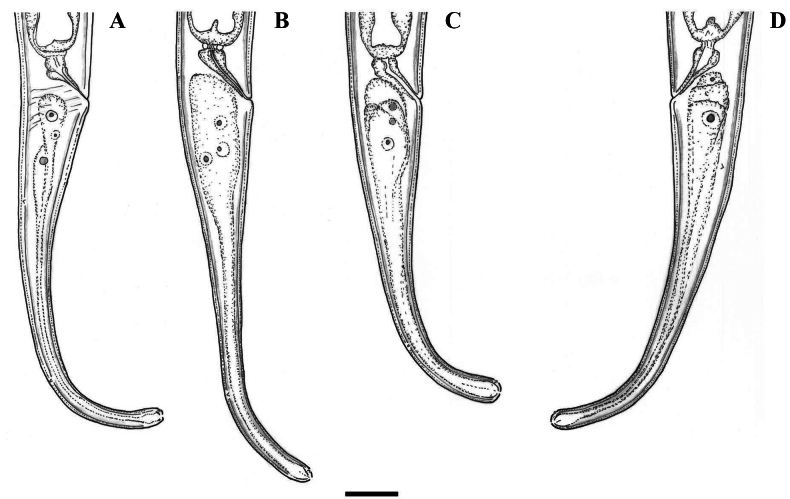
Line drawings of the tail region in females of *Mononchuspseudoaquaticus* sp. nov. from populations collected in Komluka Island (**A**) and riverbanks of the rivers Veleka (**B**), Maritsa (**C**) and Danube (**D**). Scale bar: 25 µm.

**Figure 6.**
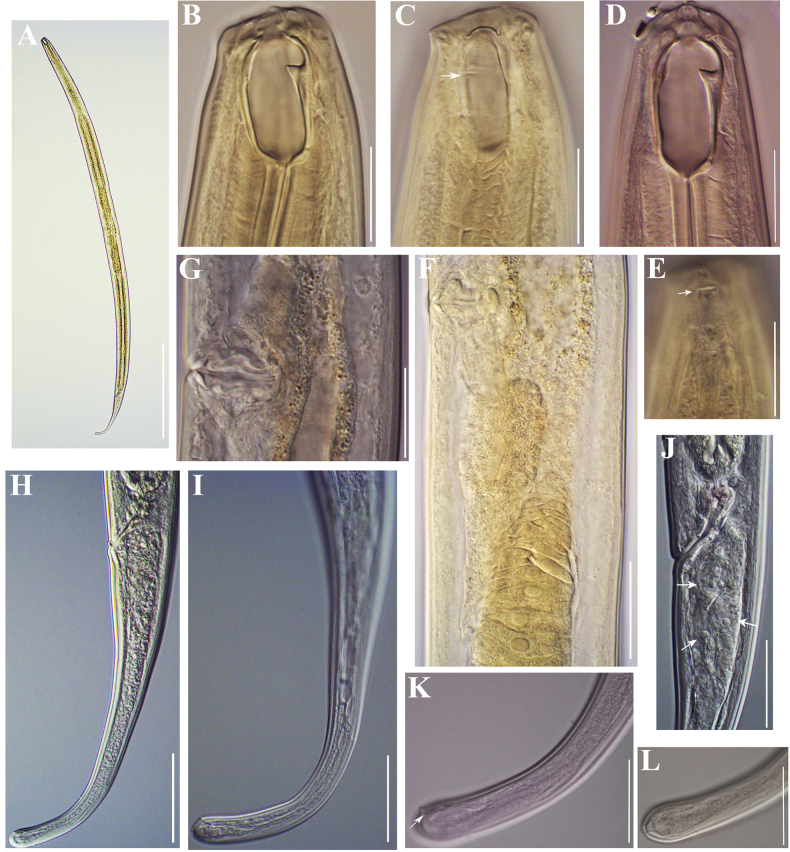
Photomicrographs of *Mononchuspseudoaquaticus* sp. nov. Holotype (**A–C, E, F, H, I, L)** and paratype (**D, G, J, K**) females: **A** body, total view **B–E** anterior region (transverse ridge arrowed in **C**; amphid opening arrowed in **Е**) **F, G** vulval region showing *pars refringens vaginae* and posterior genital branch (**F**) **H–J** tail (caudal glands arrowed in **J**) **K, L** tail tip showing one small papilla (arrowed in **K**) and terminal spinneret (**L**). Scale bars: 400 µm (**A**); 20 µm (**B–E, G, J, K, L**); 30 µm (**F, I, H**).

**Figure 7.**
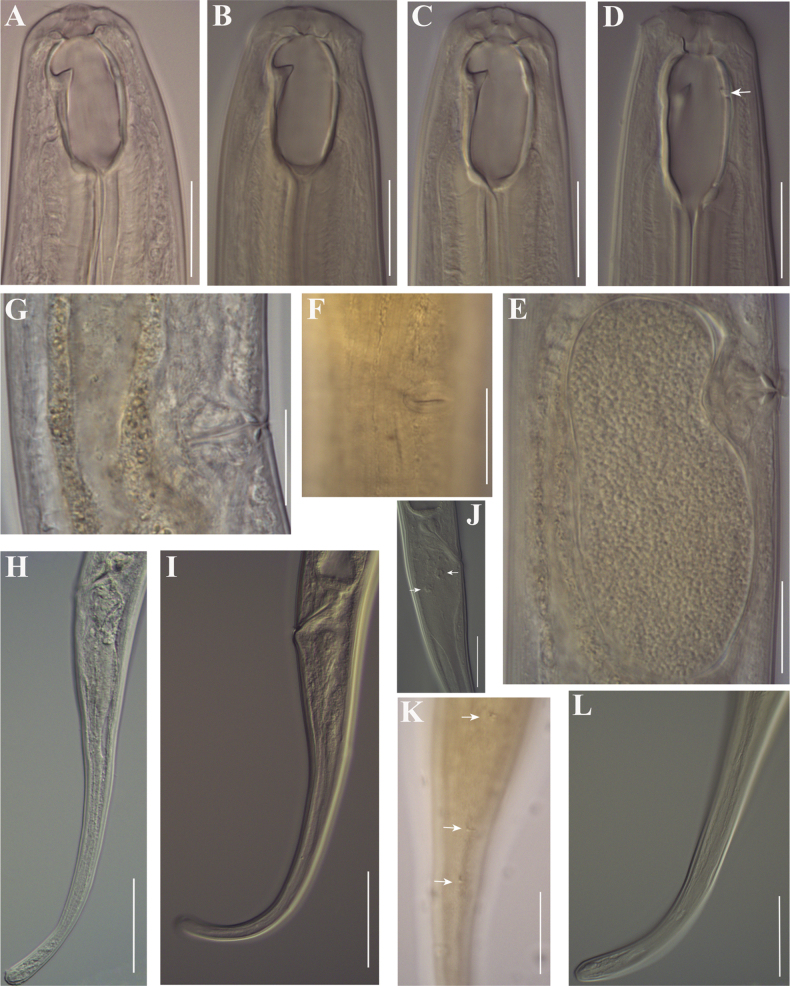
Photomicrographs of *Mononchuspseudoaquaticus* sp. nov. Females from populations collected from riverbanks of the rivers Veleka (**A, G, H**), Danube (**B, D, E, I, J**), Shirokoleshka (**C, L**) and Maritsa (**F**, **K**): **A–D** anterior region (transverse ridge arrowed in **D**) **E–G** vulval region showing an egg (**E)** vulval opening, subventral view (**F**) and *pars refringens vaginae* (**G**) **H, I** tail **J** caudal glands (arrowed) **K** caudal pores (arrowed) **L** tail tip. Scale bars: 20 µm (**A–G, K**); 30 µm (**J, L**); 50 µm (**H, I**).

Figs 3 , 4 , 5 , 6 , 7

Mononchus aquaticus *sensu*Lazarova et al. (2004) (Syn.)
Mononchus sp. 1 *sensu*Mejía-Madrid (2018) (Syn.)

###### Description.

**Female** [Based on 4 specimens from the type-population and 8 voucher specimens from other populations; see Table 3 for measurements.] Body slender (*a* = 20.2–33.6), almost straight; body diameter at mid-body 44–71. Cuticle smooth under light microscope, 2–2.5 thick along body, 3–3.5 thick in post-anal region. Lip region rounded, almost continuous with adjoining body, 2.4–3.7 as wide as high; papillae small, conical; cephalic papillae somewhat larger than labial. Body at posterior end of pharynx 1.8–2.5 times as wide as body width at lip region. Amphids caliciform, with oval apertures, 4 ± 0.5 (3.5–5.0; *n* = 10), at 8–12 from anterior end; amphid position varying from little anterior to tooth apex to level of anterior end of buccal capsule. Buccal capsule elongate-oval, slightly flattened at base, about twice as long as wide (1.8–2.0; *n* = 10), 1.2–1.3 times as long as the labial diameter; its ventral wall 2–3 thick, dorsal wall posterior to dorsal tooth 3–4 thick. Dorsal tooth strong, its anterior margin 4 ± 0.5 (3–5) wide, located at 6 ± 0.4 (5–6.5) from anterior end of buccal capsule, its anterior margin perpendicular to vertical plane. Buccal capsule with short transverse ridge, small tooth-like projection visible in some specimens in sublateral position (*n* = 2). Ventro-sublateral transverse ribs of buccal capsule weak, situated just posterior to tooth apex. Nerve-ring at 108 ± 8 (96–125) from anterior end of body. Excretory pore small, not well visible, at level of posterior margin of nerve-ring. Reproductive system amphidelphic. Anterior genital branch 171 ± 35 (116–226) long, posterior genital branch 166 ± 32 (120–205) long. Ovaries well developed, anterior ovary 105 ± 39 (65–125; *n* = 11) long, posterior ovary 106 ± 26 (70–135; *n* = 11) long. Oviduct with well-marked *pars dilatata oviductus*, 20–30 wide. Uterus a short tube with thick walls, 25–35 long. Vagina slightly swollen, with straight walls, its length representing 28–38% of corresponding body width; *pars refringens vaginae* as two smooth rhomb-shaped sclerotised pieces 3–6 long and 2–3 wide. Two females were recovered possessing a single large, thin-shelled uterine egg measuring 86–94 × 37–46 (specimens from River Maritsa and Komluka Island). Vulva a transverse slit. Vulva-anus distance equals 2.9–4.2 tail lengths. Tail long, slender, initially conoid, then almost cylindrical (10–13 wide) and slightly swollen at the tip, slightly curved ventrally in the third part; tail length represents 10–14% of body length. Caudal glands moderately developed, arranged in group. Tail tip rounded, with terminal spinneret and one small papilla. One female with abnormal tail, very short and almost straight. **Male.** Not found.

**Table 3.** Morphometric data for females of *Mononchuspseudoaquaticus* sp. nov. collected in five riparian localities in Bulgaria.

Locality	Vetren, Silistra Province		Komluka Island	Shiroka Lakа, Smolyan Province	Brodilovo, Burgas Province	Near Plovdiv, Plovdiv Province^a^
River	Danube (Southern Dobruja)		Danube	Shirokoleshka (Rhodope Mountains)	Veleka (Strandzha Mountains)	Maritsa (Upper Thracian Plain)
Habitat	*Salix* sp. (soil)		*Populus* sp. (soil)	*Salix* sp. (soil)	*Alnusglutinosa* (soil)	*Salix* sp. (soil)
*n*	Holotype	Paratypes (*n* = 3)	(*n* = 2)	(*n* = 1)	(*n* = 3)	(*n* = 2)
*L* (mm)	1.45	1.52, 1.60, 1.23	1.72, 1.88	1.61	1.60, 1.71, 1.69	1.81, 1.50
*a*	20.2	28.7, 32.0, 28.0	27.7, 33.6	30.9	27.5, 33.5, 28.6	28.3, 29.4
*b*	4.0	4.5, 4.5, 4.0	4.6, 4.5	4.4	4.4, 4.6, 4.7	4.6, 4.5
*c*	7.5	–, 8.4, 7.2	8.5, 9.1	8.9	7.8, 8.3, 8.0	10.2, –
*c*’	5.0	–, 5.8, 5.7	5.3, 5.8	5.3	5.1, 5.8, 5.4	4.7, –
*V* (%)	48.3	50.7, 49.7, 53.9	49.7, 50.0	50.7	50.8, 50.3, 48.4	48.8, 50.9
*G1* (%)	12.9	9.9, 9.7, 9.4	12.8, 11.7	9.1	10.1, 8.0, 9.9	12.5, 11.3
*G2* (%)	13.3	13.3, 10.3, 9.9	11.5, 9.9	8.7	7.5, 7.5, 10.0	11.3, 11.1
Buccal capsule length	29	31, 31, 29	29, 33	32	30, 30, 29	31, 30
Buccal capsule width	16	16, 16, 15	15, 16	16	15, 16, 16	16, –
Tooth apex from anterior end of buccal capsule	6	7, 6, 5	6, 7	7	5, 6, 6	6, 6
Position of tooth apex (%)^b^	21	21, 19, 18	19, 20	20	18, 20, 21	19, 20
Excretory pore from anterior end	118	121, 121, 112	–	126	129, 131, 124	153, 107
Nerve-ring from anterior end	96	102, 106, 99	108. 125	114	109, 111, 101	117, 110
Pharynx length	365	342, 359, 305	371, 420	369	364, 372, 355	392, 335
Lip region height	7	10, 8, 8	8, 10	8	8, 9, 8	9, 8
Lip region width	25	24, 24, 23	25, 26	26	24, 25, 23	26, 24
Amphid from anterior end	9	11, 11, 9	8, 10	12	10, 12, 11	–
Body diameter at pharynx base	62	49, 50, 43	50, 49	48	52, 49, 52	56, 47
Maximum body diameter	72	53, 50, 44	62, 56	52	58, 51, 59	64, 51
Body diameter at mid-body	71	53, 49, 44	59, 52	50	58, 50, 59	63, 51
Body diameter at vagina	72	50, 50, 44	62, 56	52	58, 51, 56	64, 50
Body diameter at anus	39	34, 33, 30	38, 36	34	40, 36, 39	38, 33
Anterior genital branch length	187	151, 155, 116	220, 220	146	162, 137, 167	226, 170
Posterior genital branch length	194	203, 165, 122	197, 186	140	120, 128, 168	205, 167
Anterior ovary length	124	94, 65, –	193, 140	85	70, 86, 79	135, 77
Posterior ovary length	135	109, 95, –	133, 135	82	70, 85, 71	130, 117
Vagina length	20	19, 18, 15	–, 16	17	19, 18, 16	19, –
Rectum length	26	28, 31, 29	28, 30	26	28, 26, 28	29, 28
Tail length	195	–, 191, 171	201, 207	180	204, 207, 210	177, –

^a^ Material reported as *M.aquaticus* by Lazarova et al. (2004). ^b^ Distance from tooth apex to anterior end of buccal capsule as % of buccal capsule length from its anterior end.

###### Type habitat and locality.

Soil around *Salix* sp. along River Danube at Vetren, Silistra Province, North Bulgaria (44°08'24"N, 27°01'47"E; elevation 20 m a.s.l.)

###### Other localities.

Komluka Island (River Danube), rivers Veleka, Shirokoleshka, and Maritsa (see Table 1 for details).

###### Type material.

The holotype female and one paratype female are deposited in the Nematode Collection of the Institute of Biodiversity and Ecosystem Research, Bulgarian Academy of Sciences, Bulgaria, under the accession numbers IBER-BAS NTC 105 and 106. One paratype female is deposited in the Wageningen Nematode Collection (WANECO), Wageningen, the Netherlands (WANECO accession number WT 4037), and one paratype female is deposited in the Nematode Collection of the U.S. Department of Agriculture (USDA), Beltsville, Maryland, USA (USDA accession number T-8065p).

###### Voucher material.

Eight voucher specimens are deposited in the Nematode Collection of the Institute of Biodiversity and Ecosystem Research, Bulgarian Academy of Sciences, Bulgaria, under the accession numbers IBER-BAS NC 5/2, IBER-BAS NC 18/3, IBER-BAS NC 16/6, IBER-BAS NC 18/5, IBER-BAS NC 78/1, IBER-BAS NC 80/1. Photovouchers for the sequenced specimens are provided in Suppl. material 1: fig. S4.

###### Representative DNA sequences.

28S rRNA gene (GenBank: PP768893 and PP768894); 18S rRNA gene (PP768902).

###### Etymology.

The species is named *Mononchuspseudoaquaticus* because of its similarity with *M.aquaticus*, hence the prefix *pseudo*- meaning false.

###### Differential diagnosis and relationships.

Females of *M.pseudoaquaticus* sp. nov. are characterised and distinguished from the congeners by a combination of features: a medium-sized body (1.23–1.88 mm); an elongate-oval, slightly flattened at the base buccal capsule measuring 29–33 × 15–16 µm, 1.8–2.0 as long as wide and distinctly shorter than 2 labial diameters (1.2–1.3 times as long as the labial diameter); amphid openings located from slightly anterior to dorsal tooth apex to level of anterior end of buccal capsule; a strong dorsal tooth situated at 18–21% of buccal capsule length from its anterior end, its anterior margin being perpendicular to the vertical plane; subventral transverse ribs located just posterior to dorsal tooth apex; didelphic (amphidelphic) reproductive system with *pars refringens vaginae* distinctly sclerotised in the form of two smooth rhomb-shaped pieces; tail (171–210 µm long, *c* = 7.2–10.2, *c*’ = 4.7–5.8) slightly curved at its posterior third, spinneret terminal.

Morphologically, *Mononchuspseudoaquaticus* sp. nov. appears most similar to *M.aquaticus*, *M.pulcher* Andrássy, 1993, and *M.caudatus* Shah & Hussain, 2016. However, *M.aquaticus* likely represents a composite species (see also Baqri and Jairajpuri 1972) based on the wide ranges of morphometric variation reported in the literature (see comparative data in Suppl. material 2: table S2). However, it is not possible to revise the identification of these materials because in many cases the findings are not documented properly and important characters such as vaginal characteristics (the shape of *pars refringens vaginae* in particular), buccal capsule shape and length/width ratio, etc., are not described, and the voucher material is inaccessible. Therefore, the species concept for *M.aquaticus* (sensu stricto) used in the present comparisons is based on the original description of Coetzee (1968) and the data by Baqri and Jairajpuri (1972) who re-examined and provided metrical data for some paratypes of *M.aquaticus*. This concept was also applied by Andrássy (2011a) in the most recent key to the species of *Mononchus* (see Suppl. material 2: table S3 for details) and in the updated key to the species of *Mononchus* provided here.

The present material differs from the type material of *M.aquaticus* (Coetzee 1968; Baqri and Jairajpuri 1972) by having: a smaller buccal capsule length/width ratio (1.8–2.0 vs 2.2–2.5); a different shape of the base of the buccal capsule (flattened vs tapering); a different direction of the anterior margin of dorsal tooth (perpendicular to the vertical plane *vs* oblique); a different shape of the vaginal sclerotised pieces (*pars refringens vaginae*) (rhomb-shaped vs drop-shaped); and a longer tail (171–210 vs 94–156 µm (mean 150 µm) (Suppl. material 2: table S3).

The new species differs from *M.caudatus* by having: a different buccal capsule length/width ratio (1.8–2.0 vs 2.0–2.5); lower *a* value (20.2–33.6 vs 34–38); more anteriorly situated nerve-ring (96–125 vs 125–134 µm); different arrangement of the caudal glands (in a group vs in tandem); and shorter rectum (26–31 vs 32–36 µm) and vagina (16–20 vs 27–29 µm) (Shah and Hussain 2016; Suppl. material 2: table S3).

Differentiation from *M.pulcher* is more complicated because the original description of Andrássy (1993) is based on two, geographically largely separated populations from Chile and Hungary. However, Andrássyʼs (1993: fig. 2) illustrations indicate that he probably dealt with different species. Unfortunately, it is impossible to separate the rather incomplete metrical data since Andrássy (1993) provided pooled data for the position of dorsal tooth apex, the length of pharynx, the width of the lip region, the body diameter at mid-body, and tail length (Suppl. material 2: table S3). Still, in addition to the morphological differences (e.g., different shape of the buccal capsule and direction of the dorsal tooth), the Hungarian population is characterised by having a smaller buccal capsule length (and hence length/width ratio) and an overall smaller body length/tail length ratio (*c*). The size of the buccal capsule is a feature that varies in rather narrow ranges for a given population/species and is one of the most important differentiating characters for all mononchids. These data indicate that the Hungarian population may represent another species. However, it is impossible to identify this material given the scant data provided in Andrássy (2011a). Therefore, our comparisons are based on the morphology and metrical data for the type-population of *M.pulcher* from Chile. The new species differs from *M.pulcher* (sensu stricto) by having: a shorter (29–33 vs 35–38 µm) and narrower (15–16 vs 16–18 µm) buccal capsule; lower values for *a* (20–34 vs 35–39); anterior margin of the dorsal tooth perpendicular to the vertical plane vs oblique, vagina not spotted in its anterior part *vs* spotted, rhomb-shaped *pars refringens vaginae* vs drop-shaped; and smaller egg length (86–94 vs 98–100 µm). Additionally, the upper ranges for body length and tail length are greater in both populations of *M.pulcher* (Suppl. material 2: table S3).

##### ﻿ Mononchus truncatus

Taxon classificationAnimaliaMononchidaMononchidae

Bastian, 1865

A2BBBEAC-3913-56C1-9139-9832328C4D4C

Figs 8 , 9

###### Description.

**Female** [Based on 14 specimens from 6 localities; see Table 4 for measurements.] Body of most specimens straight, with only last part of tail ventrally curved (body C-shaped upon fixation in a few specimens), comparatively slender, body diameter at mid-body 53–71. Cuticle smooth under light microscope, 2–3 thick along most of body, thicker (4–5) in post-anal region. Lip region rounded, continuous with adjoining body, papillae small, cephalic papillae very small and rounded, labial papillae somewhat larger and conical. Body at posterior end of pharynx 1.2–1.4 times as wide as body width at lip region. Amphids with oval apertures, situated at the beginning or middle of buccal capsule, at 11 ± 1 (10–13) (*n* = 12) from anterior end and 40 ± 3 (37–44) (*n* = 12) from posterior end of buccal capsule, aperture 4.5 ± 0.5 (4–5) (*n* = 12) wide. Buccal capsule oval, tapering at base, 2.0–2.3 as long as wide or 1.3–1.7 times as long as lip region width; its ventral wall 2–3 thick, dorsal wall posterior to dorsal tooth ~ 3–5 thick. Dorsal tooth strong, its anterior margin 5 ± 0.6 (4–6) (*n* = 12) wide, located at 11 ± 0.5 (10–12) from anterior end of buccal capsule. Ventral wall with short, not so well visible rib, ventro-sublateral transverse ribs located at level of tooth apex or slightly more anterior. Nerve-ring at 127 ± 8 (116–144) (*n* = 12) from anterior end of body. Excretory pore weakly marked, posterior to nerve-ring. Reproductive system amphidelphic. Anterior genital branch 193 ± 14 (175–223) long, posterior branch somewhat longer, 204 ± 16 (187–240) long. Ovaries well developed, not reaching uterus-oviduct junction; anterior ovary 107 ± 17 (75–142) (*n* = 12) long, posterior ovary 114 ± 18 (95–146) (*n* = 12) long. Oviduct with marked *pars dilatata oviductus*, 33 ± 7 (20–45) wide. Uteri thick-walled tubes, 40–60 long, length ranges for anterior and posterior uterus almost identical. Vagina with straight walls, 28 ± 3 in length representing 24–33% of corresponding body width; *pars refringens vaginae* as two rounded drop-shaped pieces with smooth surface, 3–5 long and 2–3 wide. Vulva transverse, not protruding. Vulva-anus distance equals 2.3–3.3 tail lengths. Tail long, slender, curved ventrally in second part, length representing 11–13% of total body length, 11–13 µm wide at cylindrical part, with rounded and slightly swollen tip. Caudal glands moderately developed, arranged in group, spinneret terminal. **Male.** Not found.

**Table 4.** Morphometric data for females of *Mononchustruncatus* collected in six riparian localities in Bulgaria.

Locality	Shiroka Laka, Smolyan Province	Teshel, Smolyan Province	Near Primorsko, Burgas Province	Slivarovo, Burgas Province	Near Plovdiv, Plovdiv Province	Vetren, Silistra Province
River	Shirokoleshka (Rhodope Mountains)	Trigradska (Rhodope Mountains)	Dyavolska (Strandzha Mountains)	Rezovska (Strandzha Mountains)	Maritsa (Upper Thracian Plain)	Danube (Southern Dobruja)
Habitat	*Salix* sp. (soil)	*Salix* sp. (litter)	*Fraxinusexcelsior* (soil)	*Ulmuslaevis* (soil)	*Salix* sp. (soil)	*Salix* sp. (soil)
*n*	(*n* = 6)	(*n* = 1)	(*n* = 1)	(*n* = 3)	(*n* = 1)	(*n* = 2)
*L* (mm)	1.94 ± 1.08 (1.83–2.09)	1.89	2.06	1.77, 1.85, 1.84	1.83	1.89, 1.91
*a*	30.6 ± 2.5 (27–34)	26.5	38.9	32.1, 33.7, 29.2	33.3	33.7, 32.4
*b*	4.1 ± 0.2 (3.7–4.3)	4.0	3.9	4.0, 4.2, 4.1	4.2	4.0, 4.2
*c*	8.3 ± 0.2 (8.1–8.7)	8.6	8.2	7.8, 8.0, 8.2	8.2	8.9, 9.3
*c*’	5.5 ± 0.4 (5.0–6.0)	5.5	6.1	6.7, 6.3, 6.2	6.2	5.0, 5.0
*V* (%)	55.2 ± 1.3 (53.4–57.3)	54.0	53.9	57.6, 53.1, 53.2	52.6	54.3, 53.8
*G1* (%)	10.1 ± 0.6 (9.5–10.9)	9.6	10.0	11.3, 9.6, 10.3	10.2	9.7, 9.8
*G2* (%)	10.4 ± 0.6 (9.7–11.6)	11.4	11.6	11.8, 10.4, 10.2	10.8	9.9, 10.1
Buccal capsule length	43 ± 2 (40–44)	44	44	42, 42, 41	40	42, 42
Buccal capsule width	20 ± 1 (19–22)	21	21	18, 19, 19	19	19, 20
Tooth apex from anterior end of buccal capsule	12 ± 1 (11–12)	11	11	11, 11, 11	10	11, 12
Position of tooth apex (%)^a^	27 ± 1 (26–29)	25	25	26, 27, 27	26	26, 27
Excretory pore from anterior end	148 ± 16 (137–176) (*n* = 5)	150	–	138, 141, 142	134	156, 146
Nerve-ring from anterior end	126 ± 10 (116–144) (*n* = 5)	–	140	138, 141, 142	122	129, 129
Pharynx length	479 ± 34 (423–518)	468	525	439, 443, 446	437	468, 450
Lip region height	9 ± 1 (8–11)	9	10	10, 10, 9	10	9, 11
Lip region width	29 ± 1 (28–30)	26	30	26, 26, 25	26	27, 25
Amphid from base of buccal capsule	40 ± 3 (37–44)	41	44	–, 39, 39	37	41, 38
Amphid from anterior end	12 ± 1 (10–13) (*n* = 4)	10	11	–, 12, 10	13	12, 11
Maximum body diameter	64 ± 6 (55–70)	71	53	55, 55, 63	55	56, 59
Body diameter at pharynx base	58 ± 4 (51–61)	53	52	51, 55, 58	53	53, 56
Body diameter at mid-body	61 ± 4 (53–65)	71	53	55, 55, 63	55	55, 59
Body diameter at vagina	64 ± 6 (55–70)	71	53	53, 54, 60	55	56, 59
Body diameter at anus	42 ± 3 (38–47)	40	41	34, 37, 36	34	42, 41
Anterior genital branch length	196 ± 18 (175–223)	181	206	199, 179, 189	207	182, 188
Posterior genital branch length	203 ± 13 (190–220)	215	240	208, 193, 187	215	187, 193
Anterior ovary length	98 ± 15 (75–115) (*n* = 5)	125	120	108, 91, –	142	107, 107
Posterior ovary length	106 ± 16 (95–135) (*n* = 5)	125	146	100, 109, –	141	101, 120
Vagina length	17 ± 1 (15–17)	17	14	–, 18, 17	17	17, 16
Rectum length	31 ± 1 (29–33)	36	27	29, 32, 31	28	32, 30
Tail length	234 ± 11 (225–254)	218	252	227, 232, 224	211	212, 205

^a^ Distance from tooth apex to anterior end of buccal capsule as % of buccal capsule length from its anterior end.

###### Voucher material.

Ten specimens are deposited in the Nematode Collection of the Institute of Biodiversity and Ecosystem Research, Bulgarian Academy of Sciences, under the accession numbers IBER-BAS NC 5/1, IBER-BAS NC 16/1-6, IBER-BAS NC 17/1, IBER-BAS NC 18/3, IBER-BAS NC 30/13, IBER-BAS NC 311/7-9. Photovouchers for the sequenced specimens are provided in Suppl. material 1: figs S5–S7.

###### Habitats and localities.

Soil around roots of *F.excelsior*, *U.laevis*, *A.glutinosa* and *Salix* sp. and litter around *Salix* sp. along banks of the rivers Shirokoleshka, Trigradska, Dyavolska, Rezovska, Veleka, Maritsa, and Danube (see Table 1 for details).

###### Representative DNA sequences.

28S rRNA gene (GenBank: PP768890–PP768892); 18S rRNA gene (PP768901).

###### Distribution.

According to the abundant published data for materials reported as *M.truncatus*, this species appears to exhibit a worldwide distribution. However, we agree with Andrássy (2011a) who doubted that all of the records referring to M. *truncatus* concern in fact this species. In Bulgaria, *M.truncatus* has been reported from many localities but with no morphological evidence supporting identification. Andrássy (1958) recorded this species for the first time in Rila Mountains and Varna. Subsequently, *M.truncatus* was reported from the North Thracian Plain (Katalan-Gateva 1962) and Pazardzhik Province (Katalan-Gateva 1965) associated with cultivated plants. In aquatic habitats, the species has been reported in sediments from 24 rivers and three lakes (Stoichev 1996; Stoichev and Chernev 2011; Stoichev and Varadinova 2011). The present study is the first to provide morphological and morphometric data for *M.truncatus* in Bulgaria.

**Figure 8.**
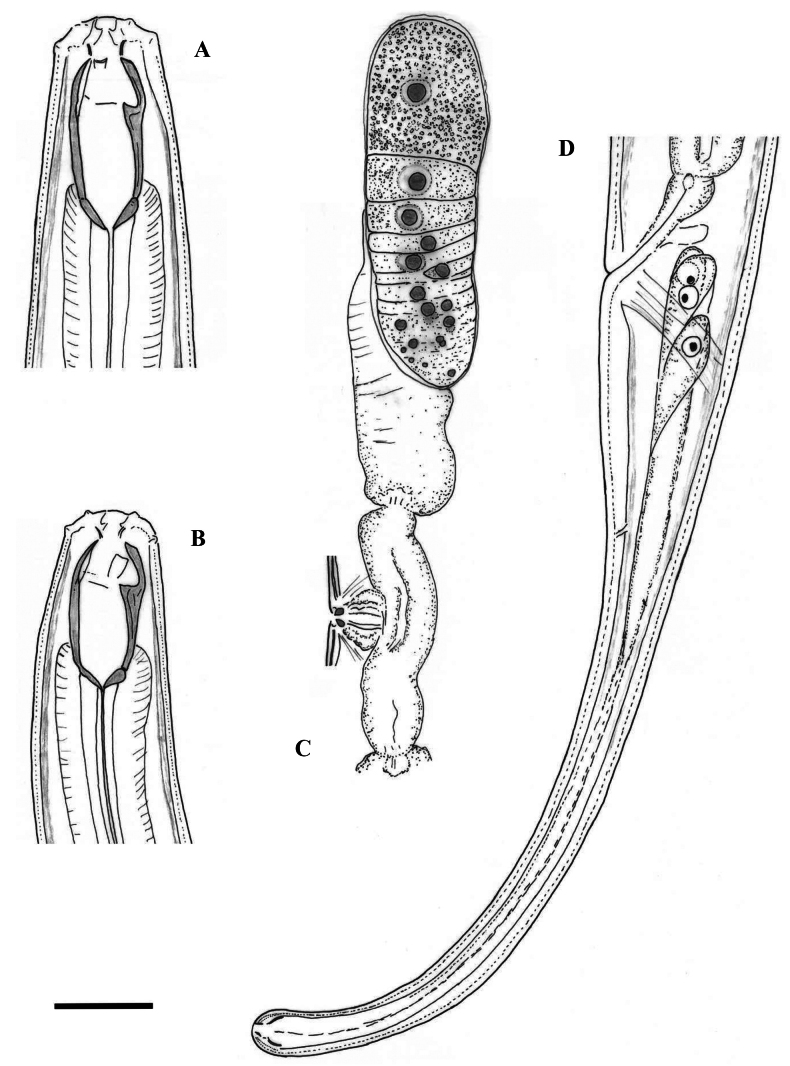
Line drawings of *Mononchustruncatus* Bastian, 1865. Females from populations collected from riverbanks of the rivers Shirokoleshka (**A, C, D**) and Maritsa (**B**): **A, B** anterior region **C** anterior genital branch **D** tail region. Scale bar: 25 µm.

###### Remarks.

Morphologically, the present material belongs to and was identified as *M.truncatus*. However, similar to the situation with *M.aquaticus* (sensu lato) considered above, *M.truncatus* also represents a composite species (Andrássy 2011a) based on the wide ranges of morphometric variation reported in the literature (see Suppl. material 2: table S4). Andrássy (2011a) summarised the data from the original description and subsequent re-descriptions of *M.truncatus* and provided novel data for a population from Hungary. This concept of *M.truncatus* (sensu stricto) (“real *M.truncatus*” of Andrássy 2011a) is applied here. Comparative morphometric data for several records deviating from this species concept are also provided in Suppl. material 2: table S4. Typically, these include studies providing data (sometimes pooled, e.g., Nakazawa 1999; Eisendle 2008) for nematodes from different localities (e.g., Botha and Heyns 1992; Nakazawa 1999; Eisendle 2008; Farahmand et al. 2009). Thus, the data by Botha and Heyns (1992) show upper ranges above the upper range (*b*, *c*, and *V*) and lower ranges below the lower range of variation in *M.truncatus* (sensu stricto) (buccal capsule width, position of tooth apex, anterior end to pharyngo-intestinal valve, body diameter at mid-body and tail length). Almost all of these differences were recorded in a single sample (Crocodile River) likely containing a misidentified specimen. Similarly, both samples studied by Farahmand et al. (2009) contain specimens with largely deviating morphometric data (Suppl. material 2: table S4).

**Figure 9.**
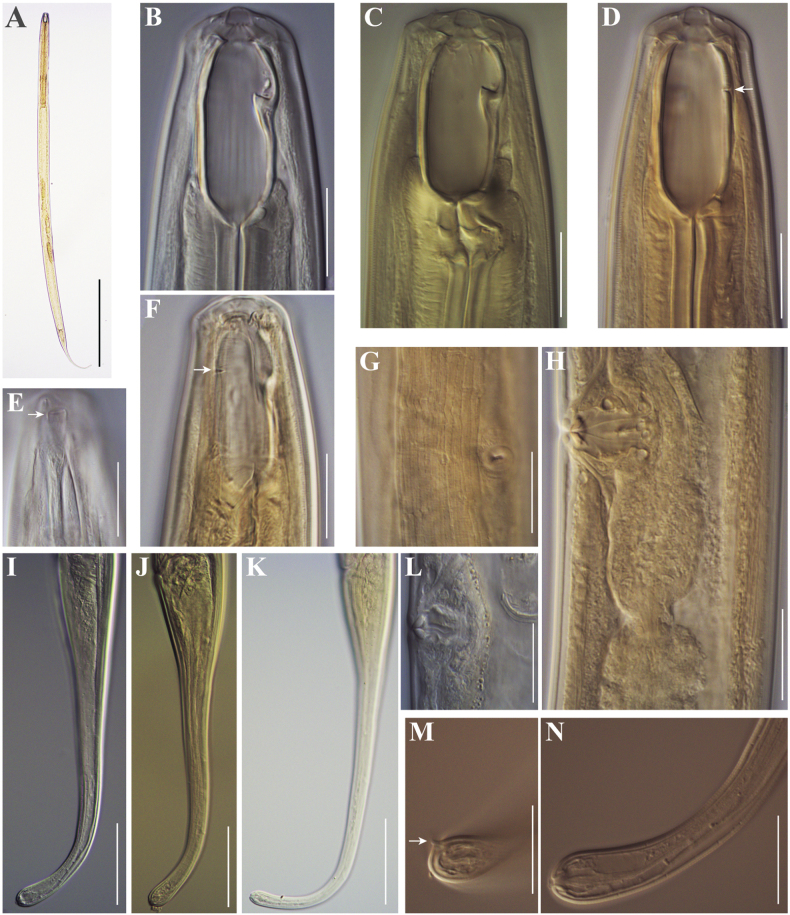
Photomicrographs of *Mononchustruncatus* Bastian, 1865. Females from populations collected from riverbanks of the rivers Rezovska (**A**, **D**, **F**, **G**, **H**, **K)**, Danube **(B**, **E**, **J**, **L**) and Shirokoleshka (**C**, **I**, **M**, **N**): **A** body, total view **B–F** anterior region (ventro-sublateral ribs arrowed in **D**; amphid opening arrowed in **E**; transverse ridge arrowed in **F**) **G, H, L** vulval region: **G** vulval opening, ventral view **H** vulva and part of posterior genital branch (posterior uterus and part of *pars dilatata oviductus*), lateral view **L** vulval region showing *pars refringens vaginae***I–K** tail **M, N** tail tip showing papilla (arrowed in **M**) and terminal spinneret (**N**). Scale bars: 400 µm (**A**); 20 µm (**B–H, L–N**); 50 µm (**I–K**).

We are also aware of two other questionable records of *M.truncatus*, not included in Suppl. material 2: table S4: Koohkan et al. (2014) reported as *M.truncatus* nematodes of a population from Ghale Asgar, Kerman Province, Iran, that do not correspond to this species because all important morphometric characters are outside the ranges of the “true” *M.truncatus* sensu Andrássy (2011a). Probably this population represents a yet undescribed species as it cannot be identified using the available keys. Similarly, Rawat and Ahmad (2000) reported *M.truncatus* as a new geographical record for India and provided a brief description based on five females. However, the measurements of some key characters such as the length of the buccal capsule (37–38 vs 42–50 μm), tail length (172–212 vs 232–283 μm) are outside the ranges of the “true” *M.truncatus* and the data provided are insufficient to identify the species. We consider that the records listed above are based on composite material.

We agree with Andrássy (2011a) who considered the description by De Bruin and Heyns (1992) to represent the “real *M.truncatus*” and add to his list of reliable records the data by Coomans et al. (1995). The present material agrees well with the characteristics of *M.truncatus* (sensu stricto) based on the original description, the description and data for the neotype population by Clark (1960), Coomans and Khan (1981), and Baqri and Jairajpuri (1972), and the description by Andrássy (2011a) except for the shorter tail (205 vs 240–283 µm) in a single specimen from Vetren (Table 4), resulting in a greater value for *c* (9.3 vs 5.8–8.6) (Suppl. material 2: tables S4, S5).

*Mononchustruncatus* was first reported from Bulgaria by Andrássy (1958) who provided limited metrical data. However, the body length reported by this author is outside the range for *M.truncatus*; additionally, two largely differing measurements (22.3 and 43.3 μm) were given for the length of the buccal capsule in two females of similar size, suggesting that this report is based on more than one species.

##### ﻿ Mononchus

Taxon classificationAnimaliaMononchidaMononchidae

sp.

A3866A6C-757C-54E7-8E69-5507E1BE2C2E

Figs 10 , 11

###### Description.

**Female** [Based on 2 females; see Table 5 for measurements]. Body slender, straight, with strongly ventrally curved tail; body diameter 42 at posterior end of buccal capsule and 45–52 at mid-body. Cuticle smooth under light microscope, 3–3.5 thick along body, thicker (4–5) around vulva and posterior to anus. Lip region rounded, continuous with adjoining body; papillae small, cephalic papillae round and somewhat more visible than labial. Body at posterior end of pharynx twice as wide as lip region. Amphids with oval apertures (5 wide), located between dorsal tooth apex and anterior end of buccal capsule. Buccal capsule oblong, with flattened base, 2.3–2.4 as long as wide or 1.7–1.8 times as long as the labial diameter, its ventral wall around 3 thick, dorsal wall posterior to dorsal tooth 4 thick. Dorsal tooth robust, its anterior margin 4 wide, located at 10–11 from anterior end of buccal capsule. Ventral wall of buccal capsule with short, not well-visible rib, transverse ventro-sublateral ribs located at level of dorsal tooth apex. Excretory pore weakly marked, posterior to nerve-ring. Reproductive system amphidelphic, genital branches short. Ovaries well developed, not reaching uterus-oviduct junction. Oviduct with well-marked *pars dilatata oviductus*, 25 wide. Uteri short, anterior uterus 30 long, posterior uterus 36 long (*n* = 1). Oviduct-uterus junction with moderately developed muscular sphincter. Vagina with straight walls and small spots next to *pars refringens vaginae*, length representing 31% of corresponding body width; *pars refringens vaginae* as two round drop-shaped sclerotised pieces with smooth surface, 4 × 2 in size; *pars distalis vaginae* well visible, ~ 4 long. Vulval opening round (Fig. 11E), vulva not protruding; vulva-anus distance equals 2.1 tail lengths. Tail long, cylindrical, strongly curved ventrad, length representing 16–18% of body length; cylindrical part of tail ~ 6 wide. Caudal glands moderately developed, arranged in tandem. Tail tip rounded, somewhat asymmetrical, dorsal part better developed, with terminal spinneret and one large setiform papilla. Three pairs of caudal pores present. **Male.** Not found.

**Table 5.** Morphometric data for females of *Mononchus* sp. and *Mononchusoblongus*.

Species	*Mononchus* sp.	*M.oblongus*
Source	Present study	Andrássy (2011a)
Locality	Waterfall “Durshin skok”, near Kopilovtsi, Montana Province	Near Ossés, South of France
River	Grafska (Balkan Mountains)	na
Habitat	*Fagussylvatica* (soil)	Liver moss (soil)
*n*	(*n* = 2)	(*n* = 6)
*L* (mm)	1.31, 1.62	1.60–1.88
*a*	27.9, 30.6	25–29
*b*	3.5, 4.1	3.5–3.7
*c*	5.7, 6.2	6.0–7.1
*c*’	7.9, 7.7	7.5–8.8
*V* (%)	51.2, 56.2	52–54
*G1* (%)	7.2, 8.3	7.6–9.4
*G2* (%)	8.0, 9.2	7.6–9.4
Buccal capsule length	45, 47	48–51
Buccal capsule width	19, 20	18–19
Tooth apex from anterior end of buccal capsule	10, 11	10.0–11.5
Position of tooth apex (%)^a^	22, 23	21–23
Excretory pore from anterior end	123, 139	–
Nerve-ring from anterior end	111, 123	–
Pharynx length	370, 392	450–504
Lip region height	8, 9	7–9
Lip region width	25, 27	22–23
Amphid from anterior end	12, 19	–^b^
Maximum body diameter	47, 53	–
Body diameter at pharynx base	47, 53	60–65
Body diameter at mid-body	45, 52	60–68
Body diameter at vagina	46, 51	–
Body diameter at anus	29, 34	30–36
Anterior genital branch length	94, 135	–
Posterior genital branch length	105, 149	–
Anterior ovary length	40, 88	–
Posterior ovary length	45, 97	–
Vagina length	–, 16	18–21
Rectum length	24, 27	–
Tail length	230, 263	264–276

^a^ Distance from tooth apex to anterior end of buccal capsule as % of buccal capsule length from its anterior end. ^b^ “Somewhat posterior to anterior end of buccal capsule.” (Andrássy, 2011a).

###### Voucher material.

Two specimens are deposited in the Nematode Collection of the Institute of Biodiversity and Ecosystem Research, Bulgarian Academy of Sciences, under the accession numbers IBER-BAS NC 316/1.

###### Habitat and locality.

Soil around roots of *F.sylvatica* near a waterfall (River Grafska, inflow of River Kopilovtsi; see Table 1 for details).

###### Remarks.

Morphologically, the specimens resemble most *Mononchusoblongus* Andrássy, 2011 regarding the shape of the buccal capsule, the actual and relative length of the tail (as percent of body length), and the position of tooth apex (Table 5; Andrássy 2011a). However, the present specimens exhibit some differences in other morphometric features and proportions such as the total body length (1.31–1.62 vs 1.60–1.88 µm), the length of the buccal capsule (45–47 vs 48–51 µm) and tail (230–263 vs 264–276 µm), the width of the lip region (25–27 vs 22–23 µm), and the ratios buccal capsule length/width (2.3–2.4 vs 2.6–2.8), buccal capsule length/lip region width (1.7–1.8 vs 2.1–2.3) and body at pharynx base/lip region width (1.9–2.0 vs 2.7–2.9).

**Figure 10.**
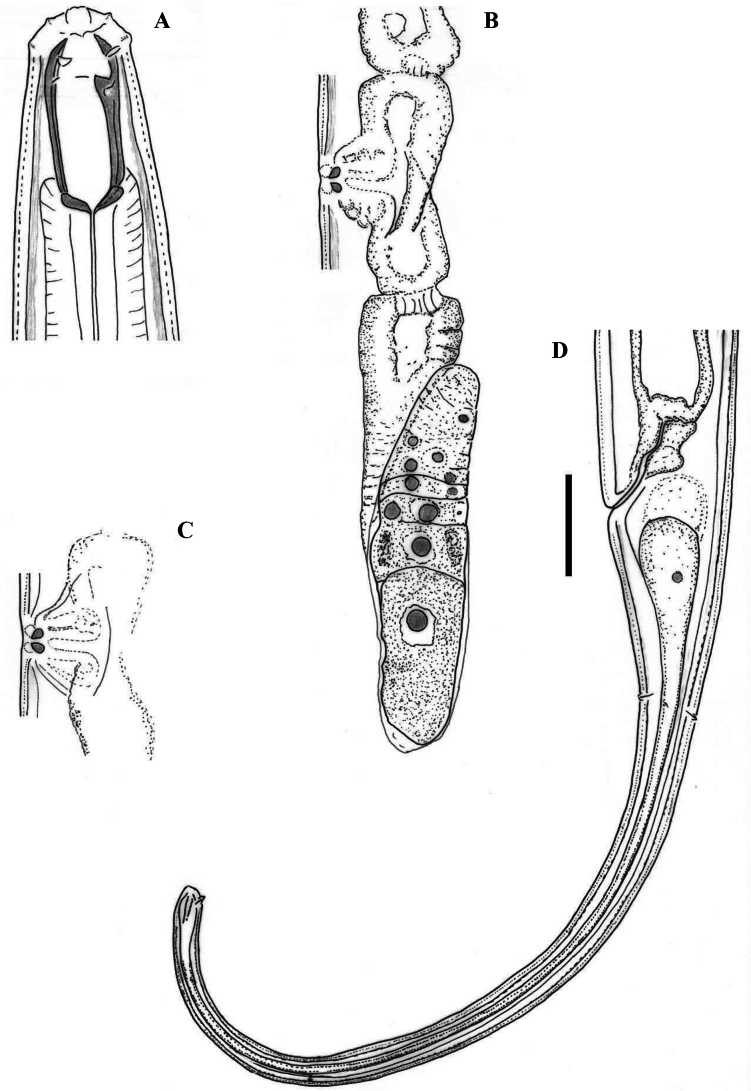
Line drawings of *Mononchus* sp. female: **A** anterior region **B** vulval region and posterior genital branch **C** vulval region **D** tail. Scale bar: 25 µm.

The present specimens also show similarities with *M.truncatus* and *M.himalayensis* Rawat & Ahmad, 2000. However, *Mononchus* sp. differs from *M.truncatus* in having a shorter body (1.31–1.62 vs 1.7–2.1 mm), a more anterior position of tooth apex (22–23 vs 25–29%), longer tail in relation to body length (16–18 vs 10–13%), smaller vulva-anus length/tail length ratio (2.1 vs 2.4–3.0), a lower *c* value (5.7–6.1 vs 7.5–8.4) and a different shape of the vulva (round vs transverse) (Andrássy 2011a). Differences between *Mononchus* sp. and *M.himalayensis* include a shorter body (1.31–1.62 vs 1.6–1.9 mm), a more anterior position of tooth apex (22–23 vs 25–31%), lower *a*- and *c*’-values (28–31 vs 33–38 and 7.7–7.9 vs 8.8–10.4, respectively) and absence of pre-vulval papilla (vs presence) (Rawat and Ahmad 2000). Probably the two females represent a species not yet described; however, additional specimens are needed to confirm the identity of the Bulgarian population.

**Figure 11.**
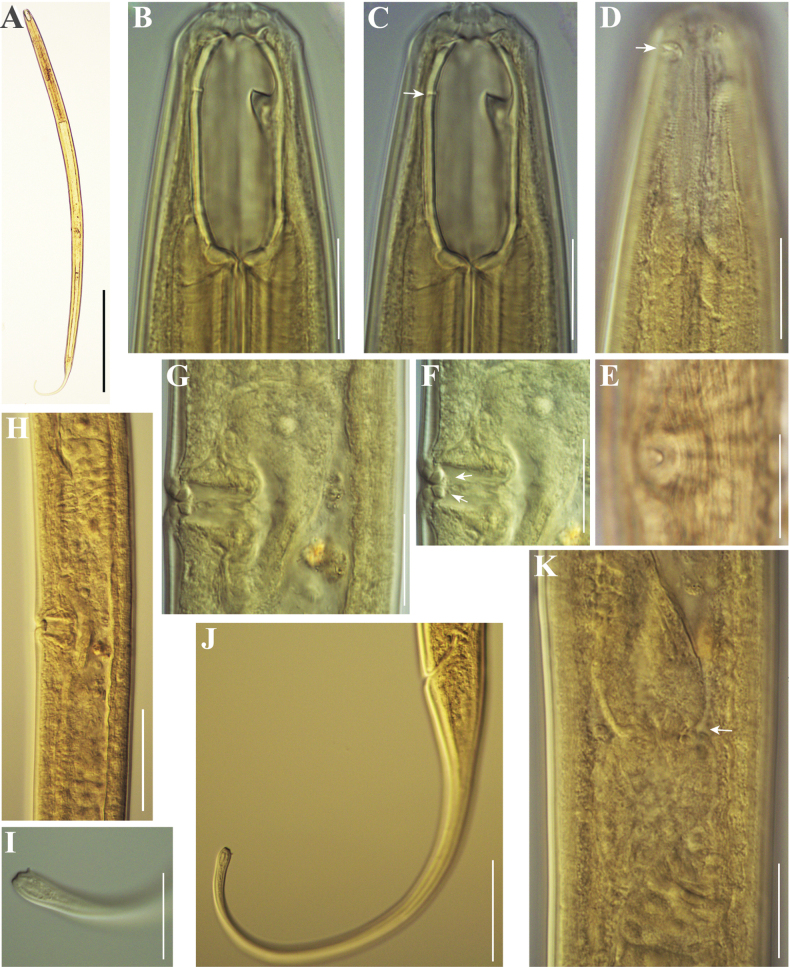
Photomicrographs of *Mononchus* sp. females: **A** body, total view **B–D** anterior region (ventro-sublateral ribs arrowed in **C**; amphid opening arrowed in **D**) **E** vulval opening, subventral view **F, G***Pars refringens vaginae* (small spots next to it arrowed in **F**) **H** reproductive system **I** tail tip **J** tail **K** sphincter of the oviduct-uterus junction (arrow). Scale bars: 400 µm (**A**); 20 µm (**B–G, I, K**); 50 µm (**H, J**).

### ﻿Key to the species of *Mononchus*

Since the last identification key to the species of the genus *Mononchus* was published by Andrássy (2011a), eight additional species have been described and one species, *M.intermedius* Tahseen & Rajan, 2009, was not considered by Andrássy (2011a) (see Table 6 for the main morphometric characters of these species). The key by Andrássy (2011a) was, therefore, modified in order to accommodate all 31 species of the genus known to date, including the new species described here. Examination of recent literature revealed that *Mononchuscaudatus* Gagarin & Naumova, 2017 was preoccupied by *Mononchuscaudatus* Shah & Hussain, 2016. Therefore, for the species described by Gagarin and Naumova (2017) we propose the replacement name *Mononchusbaikalensis* (Gagarin & Naumova, 2017) nom. nov. after the type locality, Lake Baikal.

**Table 6.** Main morphometric data for the nine additional species of *Mononchus* described after 2011 and included in the key to species.

Species	*M.amplus* Gagarin & Naumova, 2017		*M.baikalensis* (Gagarin & Naumova, 2017) nom. nov.		*M.caudatus* Shah & Hussain, 2016	*M.intermedius* Tahseen & Rajan, 2009	*M.labiatus* Shah & Hussain, 2016	*M.minutus* Naumova & Gagarin, 2018		*M.oryzae* Ishaque et al., 2022	*M.prodentatus* Shah & Hussain, 2016	*M.pseudoaquaticus* sp. nov.
*L* (mm)	♀6.74–7.24	♂6.90	♀3.35	♂ 3.35–3.72	♀1.73–1.92	♀1.32–1.65	♀1.31–1.79	♀2.38–2.89	♂2.34–2.83	♀1.51–1.53	♀1.69–1.76	♀1.23–1.88
*a*	52– 61	50	22	22–26	34–37	20.5–28.8	30–36	26–33	25–33	28.4–31.7	31–33	21–36
*b*	4.7– 4.8	5.0	3.5	3.4–3.6	4.0–5.0	3.8–4.7	3.0–4.0	3.3–3.7	3.2–3.8	3.6–3.9	4.0–5.0	4.0–4.6
*c*	11.3–11.6	16.2	9.7	11.2–13.0	9.0–10.0	8.7–10.6	7.0–8.0	12.8–15.1	14.0–16.1	12.3–13.0	8.0–9.0	7.2–10.2
*cʼ*	8.9	4.6	5.1	2.9–3.5	5.0–6.0	3.9–5.8	6.0–7.0	3.3–4.4	2.5–3.0	3.9–4.2	5.0–6.0	4.7–5.8
*V* (%)	59	–	53	–	48–51	50–55	56–64	56–61	–	59–61	53–55	48–54
Lip region width	58–60	63	50	54–60	24–25	20–26	24–25	35–40	34–38	23–24	16–17	23–26
Buccal capsule length	70–78	80	110	105–112	30–33	36–44	29–43	65–74	64–72	34–35	33–34	29–33
Position of tooth apex (%)^a^	27–29	27	30	28–30	18–21	25–30	27–35	9–14	–	19–20	24–28	18–21
Tail length	595–625	425	345	275–300	190–195	145–182	193–231	175–208	162–188	117–122	196–200	171–207
Supplements	–	40	–	31–32	–	–	–	–	21–25	–	–	–
Spicule	–	165	–	220–235	–	–	–	–	205–215	–	–	–

^a^ Distance from tooth apex to anterior end of buccal capsule as % of buccal capsule length from its anterior end. References: Gagarin and Naumova (2017); Shah and Hussain (2016); Tahseen and Rajan (2009); Naumova and Gagarin (2018); Ishaque et al. (2022).

**Table:** 

1	Large species, body 2.4–7.0 mm long	**2**
–	Smaller species, body 0.9–2.1 mm long	**13**
2	Tail very short, about 2 anal body diameters long	**3**
–	Tail longer, (3–) 4–9 anal body diameters long	**6**
3	Posterior third of tail digitate, ventrally curved	***M.mulveyi* Andrássy, 1985**
–	Posterior third of tail not digitate, more or less straight	**4**
4	Buccal capsule 100–120 μm long, nearly 3 times as long as wide	***M.tajmiris* Gagarin, 1991**
–	Buccal capsule 50–90 μm long, about twice as long as wide	**5**
5	Buccal capsule 80–90 μm long; spicule 300 μm long	***M.angarensis* Gagarin, 1984**
–	Buccal capsule about 50 μm long; spicule 120 μm long	***M.maduei* Schneider, 1925**
6	Body 5.0–7.2 mm long	**7**
–	Body 2.4–3.7 mm long	**9**
7	Body 5.0–6.4 mm long; tail as long as 5–6 (♂♂ 2.4) anal body diameters	***M.superbus* Mulvey, 1978**
–	Body 6.7–7.2 mm long; tail as long as 9 (♂♂ 4.6) anal body diameters	***M.amplus* Gagarin & Naumova, 2017**
9	Buccal capsule > 80 μm long;	**10**
–	Buccal capsule 46–74 μm long;	**11**
10	Buccal capsule 80–84 μm long; tail as long as 3–4 anal body diameters	***M.agilis* Gagarin & Mataphonov, 2004**
–	Buccal capsule 105–112 μm long; tail as long as 5 (♂♂ 2.9–3.5) anal body diameters	***M.baikalensis* (Gagarin & Naumova, 2017) nom. nov.**
11	Dorsal tooth apex at up to 16% of buccal capsule length from its anterior end; tail as long as 3–6 anal body diameters	**12**
–	Dorsal tooth apex at 28–30% of buccal capsule length from its anterior end; tail as long as 8–9 anal body diameters	***M.altiplanicus* Andrássy, 2011**
12	Body 2.8–3.5 mm long; buccal capsule 46–56 × 20–25 μm; spicules relatively short (134–140 μm)	***M.niddensis* Skwarra, 1921**
–	Body 2.4–2.9 mm long; buccal capsule 65–74 × 28–31 μm; spicules longer (205–215 μm)	***M.minutus* Naumova & Gagarin, 2018**
13	Monodelphic species	***M.italicus* Andrássy, 1959**
–	Didelphic species	**14**
14	Tail quite short (as long as 1.5–2 anal body diameters); spinneret subdorsal	***M.clarki* Altherr, 1972**
–	Tail as long as 3 anal body diameters or longer (*c*’ = up to 15); spinneret terminal	**15**
15	Buccal capsule small, 18–23 μm long	**16**
–	Buccal capsule larger, 26–50 μm long	**17**
16	Buccal capsule very narrow (nearly 3 times as long as wide); dorsal tooth apex quite close to the anterior end of buccal capsule	***M.tunbridgensis* Bastian, 1865**
–	Buccal capsule wider (twice as long as wide); dorsal tooth apex at 28–33% of buccal capsule length from its anterior end	***M.loofi* Winiszewska, 1998**
17	Tail as long as 7–15 (mostly 9–14) anal body diameters	**18**
–	Tail as long as 3–8 (mostly 4–7) anal body diameters	**20**
18	Tail 340–390 μm long, as long as 13–15 anal body diameters	***M.syrmatus* Andrássy, 2008**
–	Tail 220–300 μm long, as long as 8–11 anal body diameters	**19**
19	Buccal capsule 40–47 μm long; one prevulval papilla present	***M.himalayensis* Rawat & Ahmad, 2000**
–	Buccal capsule 28–35 μm long; prevulval papilla absent	***M.sandur* Eisendle, 2008**
20	*Pars refringens vaginae* not sclerotised	***M.sinensis* Soni & Nama, 1983**
–	*Pars refringens vaginae* distinctly sclerotised	**21**
21	Subventral transverse ribs located anteriorly to tooth apex	**22**
–	Subventral transverse ribs located at level of or posterior to tooth apex	**23**
22	Lip region relatively wide (24–28 μm); cylindrical portion of tail 10–12 μm thick	***M.truncatus* Bastian, 1865**
–	Lip region narrower (20 μm); cylindrical portion of tail 5–7 μm thick	***M.medius* Andrássy, 2011**
23	Amphid aperture posterior to dorsal tooth	***M.laminatus* Zullini, Loof & Bongers, 2002**
–	Amphid aperture anterior to dorsal tooth	**24**
24	Tail as long as 3–4 anal body diameters	**25**
–	Tail as long as 4–7 anal body diameters	**26**
25	Body 1.6–2.1 mm long; dorsal tooth apex at 22–24% of buccal capsule length from its anterior end; tail 176 μm	***M.nudus* Gagarin, 1991**
–	Body 1.5 mm long, dorsal tooth apex at 30–35% of buccal capsule length from its anterior end; tail 117–122 μm	***M.oryzae* Ishaque, Iqbal, Dawar & Kazi, 2022**
26	Buccal capsule two labial diameters long or longer	**27**
–	Buccal capsule conspicuously shorter than two labial diameters	**28**
27	Buccal capsule oblong, 47–50 μm long, labial diameter 22–23 μm	***M.oblongus* Andrássy, 2011**
–	Buccal capsule barrel-shaped, 33–34 μm long, labial diameter 16–17 μm	***M.prodentatus* Shah & Hussain, 2016**
28	Dorsal tooth apex at > 25% of buccal capsule length from its anterior end	**29**
–	Dorsal tooth apex at < 25% of buccal capsule length from its anterior end	**30**
29	Buccal capsule 36–44 μm long, *c* = 8.7–10.6; tail 145–182 μm long	***M.intermedius* Tahseen & Rajan, 2009**
–	Buccal capsule 29–43 μm long, *c* = 7–8; tail 193–231 μm long	***M.labiatus* Shah & Hussain, 2016**
30	Buccal capsule (33)35–38 μm long, vagina spotted in its anterior part	***M.pulcher* Andrássy, 1993**
–	Buccal capsule 29–33 μm long, vagina not spotted	**31**
31	Buccal capsule 1.8–2.0 times as long as wide, *pars refringens vaginae* rhomb-shaped	***M.pseudoaquaticus* sp. nov.**
–	Buccal capsule 2.2–2.5 times as long as wide, *pars rrefringens vaginae* drop-shaped	**32**
32	Body 1.2–1.7 mm long, rectum length 24–25 μm	***M.aquaticus* Coetzee, 1968**
–	Body 1.7–1.9 mm long, rectum length 32–36 μm	***M.caudatus* Shah & Hussain, 2016**

### ﻿Molecular phylogenies

**Figure 12.**
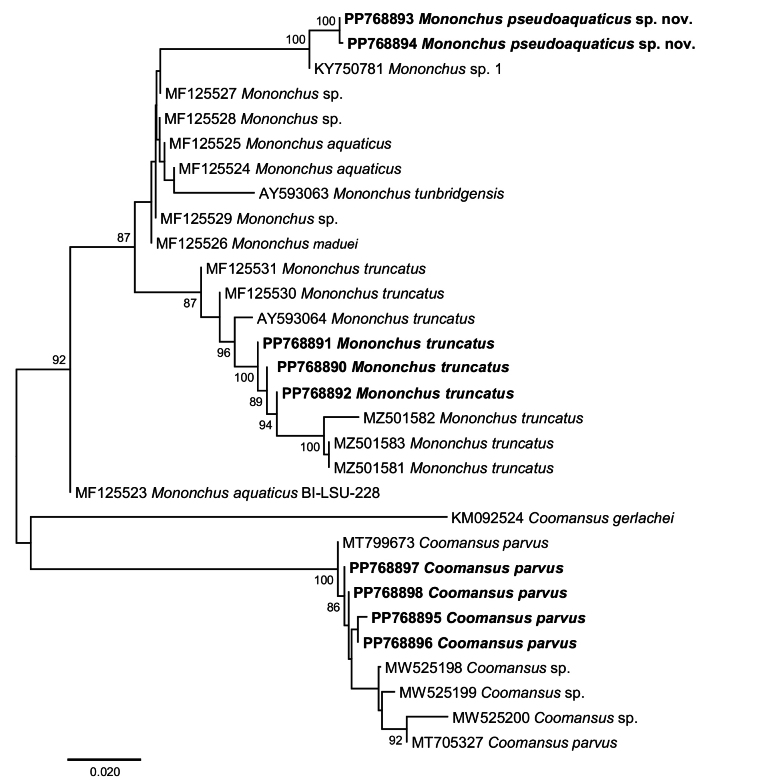
Neighbour-joining tree based on the 28S rDNA (domains D1-D3) dataset (1293 nt positions). The newly generated sequences are indicated in bold. Only bootstrap values > 70% are shown.

To assess the associations of the newly generated sequences (4 for *C.parvus* and 5 for *Mononchus* spp.) from the nematode populations sampled in Bulgaria, we carried out an exploratory neighbour-joining (NJ) analysis on an untrimmed 28S rDNA alignment (domains D1-D3), including representative sequences for *Mononchus* spp. (20 sequences) and *Coomansus* spp. (15 sequences). Using pairwise deletion of missing data allowed us to include more taxa and sequences, e.g., several sequences of Schenk et al. (2017), including sequences for *M.aquaticus*, albeit with a short overlap (GenBank codes MF-XXX, D3-D5 region) (Fig. 12). The novel isolates of *C.parvus* formed a reciprocally monophyletic clade with *C.parvus*, *C.batxatensis* Vu, 2021 and *Coomansus* spp. with maximum support; the clade of *Coomansus* spp. was recovered as sister to *C.gerlachei* (de Man, 1904) Jairajpuri & Khan, 1977 (GenBank: KM092524) but with poor statistical support. The isolates of *M.pseudoaquaticus* sp. nov. clustered with maximum support with *Mononchus* sp. 1 sensu Mejía-Madrid (2018) within the strongly supported clade of *Mononchus* spp. comprising the novel and published isolates of *M.aquaticus*, *M.truncatus*, *M.maduei*, *M.tunbridgensis*, and *Mononchus* sp. sensu Schenk et al. (2017) to the exclusion of one isolate identified as *M.aquaticus* (GenBank: MF125523; Schenk et al. 2017). This molecular prospecting analysis confirmed the identification of the novel isolates based on the detailed morphological analysis (see above) and indicated that *Mononchus* sp. 1 sensu Mejía-Madrid (2018) belongs to the new species described here.

**Figure 13.**
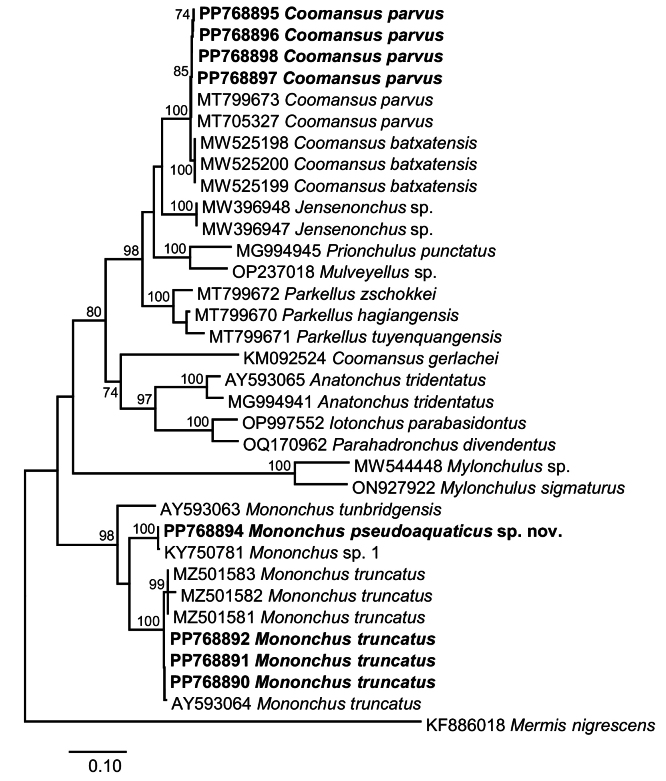
Maximum likelihood phylogeny based on the 28S rDNA (domains D2-D3) dataset (779 nt positions). The newly generated sequences are indicated in bold. Only bootstrap values > 70% are shown.

Next, we assessed the phylogenetic relationships of the novel isolates with representatives of the suborder Mononchina using two alignments. Upon trimming to the length of the shortest sequence, the 28S rDNA (domains D2-D3) alignment comprised a total of 779 nt positions and contained sequences for representatives of ten genera of the families Anatonchidae (*Anatonchus* Cobb, 1916, *Iotonchus* Cobb, 1916, *Jensenonchus* Jairajpuri & Khan, 1982, *Mulveyellus* Siddiqi, 1984 and *Parahadronchus* Mulvey, 1978), Mononchidae (*Coomansus*, *Mononchus*, *Parkellus* and *Prionchulus* Cobb, 1916) and Mylonchulidae (*Mylonchulus* Jairajpuri, 1969). There were no sequence data for *Miconchus* spp. and *Actus* spp., and the available sequences for *Clarkuspapillatus* (Bastian, 1865) Jairajpuri, 1970 (domains D3-D5) could not be used due to the very small overlap. Overall, the topology of the ML tree (Fig. 13) was well resolved with two strongly supported main clades: (i) *Mononchus* spp. (98% supported); and (ii) a large clade (80% supported) comprising the remaining genera except for *Mylonchulus*. Within the *Mononchus* clade, the novel sequences for *M.truncatus* clustered with four published sequences for *M.truncatus* with maximum support and *M.pseudoaquaticus* sp. nov. clustered with a sequence for *Mononchus* sp. 1 sensu Mejía-Madrid (2018), again with maximum support. The second clade had fully resolved internal topology with two large sub-clades, one (100% supported) comprising *Coomansus* spp. (*C.parvus* + *C.batxatensis*) plus representatives of *Jensenonchus*, *Prionchulus*, *Mulveyellus*, and *Parkellus*, and one (74% supported) comprising *C.gerlachei* and representatives of *Anatonchus*, *Iotonchus* and *Parahadronchus*. The relationships of the strongly supported (100%) clade of *Mylonchulus* spp. remained unresolved.

**Figure 14.**
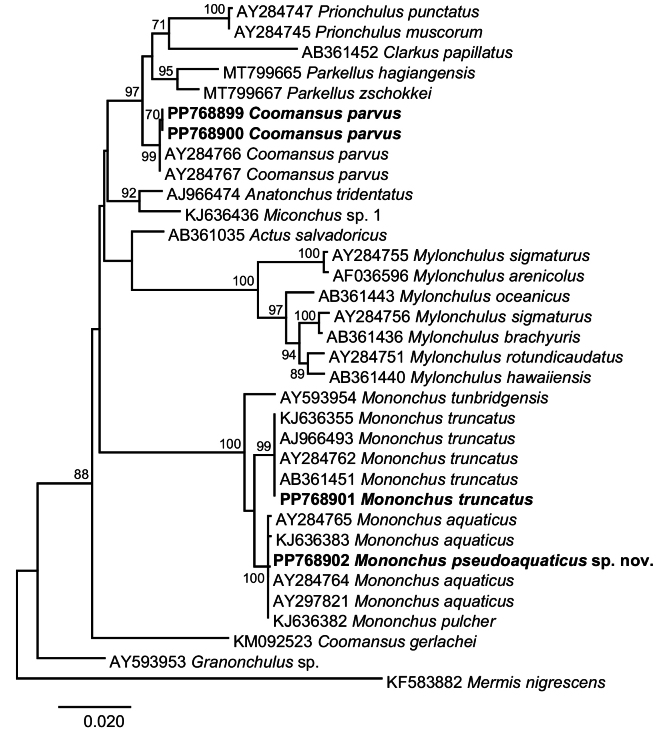
Maximum likelihood phylogeny based on the 18S rDNA dataset (1636 nt positions). The newly generated sequences are indicated in bold. Only bootstrap values > 70% are shown.

The 18S rDNA alignment comprised a total of 1636 nt positions after trimming the ends to match the shortest aligned sequences and contained sequences for representatives of ten genera of the families Anatonchidae (*Anatonchus* and *Miconchus* Andrássy, 1958), Mononchidae (*Actus* Baqri & Jairajpuri, 1974, *Clarkus* Jairajpuri, 1970, *Coomansus*, *Mononchus*, *Parkellus*, and *Prionchulus*) and Mylonchulidae (*Granonchulus* Andrássy, 1958 and *Mylonchulus*). The available sequences for representatives of the genera *Iotonchus*, *Jensenonchus*, *Mulveyellus*, and *Parahadronchus* were excluded from the analyses because they were too short and did not exhibit sufficient overlap with the alignment. The topology of the ML tree (Fig. 14) exhibited poorly resolved basal nodes and four strongly supported clades: (i) *Mononchus* spp. (100% supported) (*M.tunbridgensis* + *M.truncatus* + *M.aquaticus* + *M.pseudoaquaticus* sp. nov. + *M.pulcher*); (ii) the remaining mononchids (*Coomansus* + *Clarkus* + *Parkellus* + *Prionchulus*) (97% supported); (iii) *Mylonchulus* (100% supported); and (iv) *Anatonchus* + *Miconchus* (both Anatonchidae) (92% supported).

At the species level, both phylogenies (18S rDNA and 28S rDNA) supported (i) the identification based on morphology of the novel isolates of *M.truncatus* and *C.parvus* both forming strongly supported reciprocally monophyletic clades, and (ii) the exclusion of *C.gerlachei* from *Coomansus*; this species was recovered as a sister taxon (74% supported) to the representatives of the Anatonchidae in the 28S rDNA phylogeny and as a basal taxon to the remaining taxa except for *Granonchulus* in the 18S rDNA analysis. However, in contrast to the clear delineation of *M.pseudoaquaticus* sp. nov. (100% supported) in the 28S rDNA phylogeny, the 18S rDNA phylogeny did not provide support for the delimitation of *M.aquaticus* (as *M.pulcher* sensu van Megen et al. 2009; GenBank: KJ636382) and *M.pseudoaquaticus* sp. nov.

At the generic level, both phylogenies recovered the three genera represented by two or more species (i.e., *Mononchus*, *Mylonchulus*, and *Parkellus*) as monophyletic with strong support. At the suprageneric level, both phylogenies resolved fewer relationships due to the small number of taxa (10 genera, 5 genera in common) and perhaps the much poorly resolved basal nodes in the 18S rDNA phylogeny. Both phylogenies recovered the Mononchidae as paraphyletic with *Mononchus* placed in a separate basal clade and Mylonchulidae and Anatonchidae nested within the second clade of the Mononchidae despite the different composition of the taxa included in the analyses. However, this is the only concordant result for the two molecular markers. Thus, the Mylonchulidae (represented by *Mylonchulus* alone) was recovered as monophyletic in the 28S rDNA phylogeny but as polyphyletic in the 18S rDNA phylogeny (represented by *Mylonchulus* and *Granonchulus*). Similarly, the Anatonchidae was monophyletic in the 18S rDNA phylogeny (2 genera: *Anatonchus* and *Miconchus*) but paraphyletic in the 28S rDNA phylogeny containing five genera, with *Anatonchus* + *Iotonchus* + *Parahadronchus* recovered in a strongly supported clade (97% supported) and *Jensenonchus* and *Mulveyellus* nested within one of the clades of the Mononchidae.

### ﻿Comparative sequence analysis

The trimmed alignments of 28S rDNA and 18S rDNA allowed a comparative assessment of the genetic divergence at the level of species (intraspecific) and genus (interspecific) as well as between genera (intergeneric) based on pairwise comparisons. As shown in Table 7, the divergence levels for 18S rDNA are much lower for all three categories of comparisons: up to 10-fold for intraspecific variation, up to ~ 4-fold for interspecific variation, and up to ~ 5-fold for intergeneric variation. The interspecific divergence in 18S rDNA sequences for *M.pulcher* sensu van Megen et al. (2009; GenBank: KJ636382), *M.aquaticus*, and *M.pseudoaquaticus* sp. nov. was particularly low (0–1 nt positions; 0–0.1%).

**Table 7.** Genetic divergence estimated for the 18S rDNA and 28S rDNA sequence pairs within and between species and between species of different genera compared in this study.

Divergence	Taxa	18S rRNA gene		28S rRNA gene	
		Differences (nt)	p-distance (%)	Differences (nt)	p-distance (%)
Intraspecific	*Mononchustruncatus*	0	0	0–7^a^	0–1.1^a^
	*Mononchusaquaticus*	0–2^b^	0–0.1^b^	1	0.2
	*Coomansusparvus*	0–1	0–0.1	0–2	0–0.3
Interspecific	*M.truncatus* vs *M.aquaticus*	13–14	0.8–0.9	59–70	8.9–10.5
	*Mononchus* spp.	13–23^b^	0.8–1.4^b^	59–77	8.9–11.8
	*Coomansus* spp.	70–71^c^	4.3–4.4^c^	6–8^d^	0.9–1.2^d^
	*Parkellus* spp.	24	1.5	34–54	5.1–8.2
	*Mylonchulus* spp.	3–52	0.2–3.2	69	10.1
Intergeneric	*Mononchus* spp. vs *Coomansus* spp.	76–80	4.7–4.9	140–158^d^	21.2–22.8^d^
	*Coomansus* spp. vs *Parkellus* spp.	27–35^e^	1.7–2.2^e^	64–74^d^	9.7–11.2^d^

^a^*M.truncatus* (GenBank: MZ501582; unpublished) excluded from the comparison (differs from the remaining isolates of *M.truncatus* at 8–15 nt positions, i.e., 1.2–2.0%). ^b^*M.pulcher* (GenBank: KJ636382) included in the comparison (differs from *M.aquaticus* at 0–1 nt positions, i.e., 0–0.1%). ^c^ Genetic divergence between *C.parvus* and *C.gerlachei*. ^d^*C.gerlachei* (GenBank: KM092524) excluded from the comparison (differs from the remaining *Coomansus* spp. at 135–137 nt positions, i.e., 20.6–20.8%). ^e^*C.gerlachei* (GenBank: KM092523) excluded from the comparison.

Comparative sequence analyses also provided support for the position in the phylogenies of the isolate identified as *C.gerlachei* (GenBank: KM092523 and KM092524) by Elshishka et al. (2015). This isolate differed from *C.parvus* at 70–71 nt positions (4.3–4.4%; 18S rDNA) and from the remaining *Coomansus* spp. at 135–137 nt positions (20.6–20.8%; 28S rDNA), values well above the genetic divergence between congeners. The isolates identified by Kagoshima et al. (2019) (GenBank: LC457639–LC457644; LC457655–LC457661) were found to be associated with high support with the isolate of Elshishka et al. (2015) that also represented the best BLAST hit for all isolates.

Genetic divergence estimates in 28S rDNA also indicated that *C.batxatensis* may be conspecific with *C.parvus* (difference at 6–8 nt positions, i.e., 0.9–1.2%). This difference is distinctly lower than the ranges of interspecific divergence within the genera *Mononchus*, *Coomansus*, *Parkellus* and *Mylonchulus*, i.e., 34–77 nt positions or 5.1–11.8%; Table 7). Furthermore, the otherwise unpublished isolate identified as *M.truncatus* (GenBank: MZ501582) may have been misidentified; this isolate differs from the remaining isolates of *M.truncatus* by 8–15 nt (1.2–2.0%). Finally, the intergeneric divergence between *Coomansus* spp. and *Parkellus* spp. falls within the range of interspecific divergence for both genes (Table 7) and this is in contrast with both model-based phylogenies supporting the distinction of *Parkellus* spp. at the generic level (Figs 13, 14).

## ﻿Discussion

To the best of our knowledge, the present study is the first to apply an integrative taxonomic approach to the diversity of mononchid nematodes in European riparian ecosystems. Our extensive, focused sampling in a range of riverine habitats in Bulgaria revealed a wide geographical distribution and altitudinal ranges of three species of the family Mononchidae of which one represents a species new to science; these were also associated with a range of tree species of seven genera (*Alnus*, *Carpinus*, *Fagus*, *Fraxinus*, *Populus*, *Salix*, and *Ulmus*). The integration of molecular and morphological data for these three species provided support for their distinct species status. Thus, our study is the first to provide taxonomically verified 18S rDNA and 28S rDNA sequences for *C.parvus*, *M.truncatus* (sensu stricto), and *M.pseudoaquaticus* sp. nov.

At the species level, phylogenetic analyses revealed that the newly sequenced isolates of *M.truncatus* (sensu stricto) and *C.parvus* consistently clustered together with published sequences for these species irrespective of the ribosomal locus (Figs 13, 14) or region of the 28S rRNA gene (Figs 12, 13). However, the 18S rDNA phylogeny did not allow delimitation of *M.pseudoaquaticus* sp. nov. and *M.aquaticus* (Fig. 14). Whereas *M.pulcher* sensu van Megen et al. (2009) (GenBank: KJ636382) not used in the analysis by these authors and by Holterman et al. (2008) is likely a misidentification, the morphological differentiation of *M.aquaticus* and *M.pseudoaquaticus* sp. nov. was strongly supported in the 28S rDNA phylogeny, suggesting that the 18S rRNA gene does not allow reliable differentiation of closely related species at least within the genus *Mononchus*. Lower resolution of the 18S rRNA gene was reported in a comparative barcoding/metabarcoding study by Schenk et al. (2020); out of 22 nematode species identified using morphology in their study, 20 species were delineated using the 28S rDNA marker and only 12 species were detected using the 18S rDNA marker. Our comparative sequence (Table 7) and phylogenetic (Fig. 14) analyses suggest that the utility of the 18S rRNA gene for species delimitation is rather limited at least for some species complexes within the genus *Mononchus*.

An alternative hypothesis for the phylogenetic results based on 18S rDNA is that the specimens of *M.aquaticus* sequenced by van Megen et al. (2009) (GenBank: KJ636382 and KJ636383), Holterman et al. (2006) (GenBank: AY284764 and AY284765) and Oliveira et al. (2004) (GenBank: AY297821) actually represent *M.pseudoaquaticus* sp. nov. However, because of the lack of sequence data for the 28S rRNA gene and deposited voucher material for these sequenced isolates, neither of these hypotheses can be tested.

We highlight that the new species described here could be clearly distinguished morphologically from *M.aquaticus* (sensu stricto) and that currently *M.aquaticus* likely represents a composite species and this may result in misidentifications of the isolates subjected to sequencing. For example, in the 28 rDNA tree of Schenk et al. (2017) the six specimens identified as *M.aquaticus* (*n* = 3), *M.maduei* and *Mononchus* sp. juv. (*n* = 3) formed a reciprocally monophyletic clade with high support (97%). However, in the present NJ analysis (Fig. 12) one isolate (GenBank: MF125523) was resolved as clearly distinct from the other two isolates of *M.aquaticus* sequenced, and in fact, from all isolates of *Mononchus* spp. (Fig. 12), thus questioning the identification of this isolate. The above considerations highlight the need for generating taxonomically verified 18S rDNA and 28S rDNA sequences for *M.aquaticus* (sensu stricto).

The isolate of *C.gerlachei* sequenced by Elshishka et al. (2015) (GenBank: KM092523 and KM092524) was not associated with the *C.parvus* clade (18S rDNA; Fig. 14) or with *C.parvus* + *C.batxatensis* clade (28S rDNA; Fig. 13) and exhibited genetic divergence levels well above the levels observed between species of the mononchid genera *Coomansus*, *Mononchus*, *Mylonchulus*, and *Parkellus* considered here (Table 7). Taken together, our comparative sequence and phylogenetic analyses strongly suggest that the isolates sequenced by Elshishka et al. (2015) and Kagoshima et al. (2019) should be distinguished at the generic level.

At the generic and suprageneric level, the present 18S and 28S phylogenies both recovered the Mononchidae as paraphyletic (as in Holterman et al. 2008 and van Megen et al. 2009). Further, a comparison with the phylogenies of the Mononchida based on 18S rDNA by Holterman et al. (2008) and van Megen et al. (2009) revealed that all four sub-clades identified by these authors (denoted M1-M4 in Holterman et al. 2008) were supported in the present phylogeny: *Mylonchulus* (sub-clade M1); *Mononchus* (sub-clade M2); *Clarkus* + *Prionchulus* + *Coomansus* (sub-clade M3); and *Anatonchus* (sub-clade M4). The differences between the present and published phylogenetic hypotheses represent (i) the recovery of the Anatonchidae as monophyletic in the present phylogeny vs paraphyletic in Holterman et al. (2008) and van Megen et al. (2009), and (ii) the lack of support for a sister-group relationship between *Mylonchulus* (sub-clade M1) and *Mononchus* (sub-clade M2) in the present phylogeny. Finally, the poorly represented Mylonchuldae (2 genera: *Mylonchulus* and *Granonchulus*) was recovered as polyphyletic in the present 18S rDNA phylogeny as in Holterman et al. (2008) and van Megen et al. (2009). These results are due to a single sequence for *Granonchulus* sp. (AY593953) by Holterman et al. (2008). Unfortunately, no voucher specimen exists to support the identification of the sequenced nematode. Further sequencing of *Granonchulus* spp., and preferably, of species from the other genera of the family, will help develop a natural hypothesis for the relationships within the Mylonchulidae and the Mononchida in general.

## Supplementary Material

XML Treatment for Coomansus parvus

XML Treatment for Mononchus pseudoaquaticus

XML Treatment for Mononchus truncatus

XML Treatment for Mononchus

## ﻿Additional information

### Author ORCIDs

Aneta Kostadinova ￼ https://orcid.org/0000-0001-7070-4968

### Data availability

All data generated or analyzed during this study are included in this published article and its supplementary files. The newly generated sequences were submitted to the GenBank database under the accession numbers PP768899–PP768902 (18S rRNA gene) and PP768890–PP768898 (28S rRNA gene).

## Supplementary materials

Supplementary material 1 Photomicrographs of sequenced specimens of *Coomansusparvus*, *Mononchuspseudoaquaticus* sp. nov., and *M.truncatus* Data type pdf https://binary.pensoft.net/file/1086401This dataset is made available under the Open Database License (http://opendatacommons.org/licenses/odbl/1.0/). The Open Database License (ODbL) is a license agreement intended to allow users to freely share, modify, and use this Dataset while maintaining this same freedom for others, provided that the original source and author(s) are credited.Stela Altash, Aneta Kostadinova, Vlada Peneva

Supplementary material 2 Comparative morphometric data for females of *Coomansusparvus*, *Mononchusaquaticus*, *M.pseudoaquaticus* sp. nov., and *Mononchustruncatus* Data type pdf https://binary.pensoft.net/file/1086402This dataset is made available under the Open Database License (http://opendatacommons.org/licenses/odbl/1.0/). The Open Database License (ODbL) is a license agreement intended to allow users to freely share, modify, and use this Dataset while maintaining this same freedom for others, provided that the original source and author(s) are credited.Stela Altash, Aneta Kostadinova, Vlada Peneva
