# Supplementary material for: ﻿Integrative taxonomic study of mononchid nematodes from riparian habitats in Bulgaria. I. Genera Mononchus Bastian, 1865 and Coomansus Jairajpuri & Khan, 1977 with the description of Mononchuspseudoaquaticus sp. nov. and a key to the species of Mononchus
Source: Zookeys. 2024 Jul 5;1206:137–80. doi: 10.3897/zookeys.1206.124237 (PMC11245642; doi:10.3897/zookeys.1206.124237)
Supplement: Supplementary material 1 — Photomicrographs of sequenced specimens of Coomansusparvus, Mononchuspseudoaquaticus sp. nov., and M.truncatus [file zookeys-1206-137_article-124237__-s001.pdf]

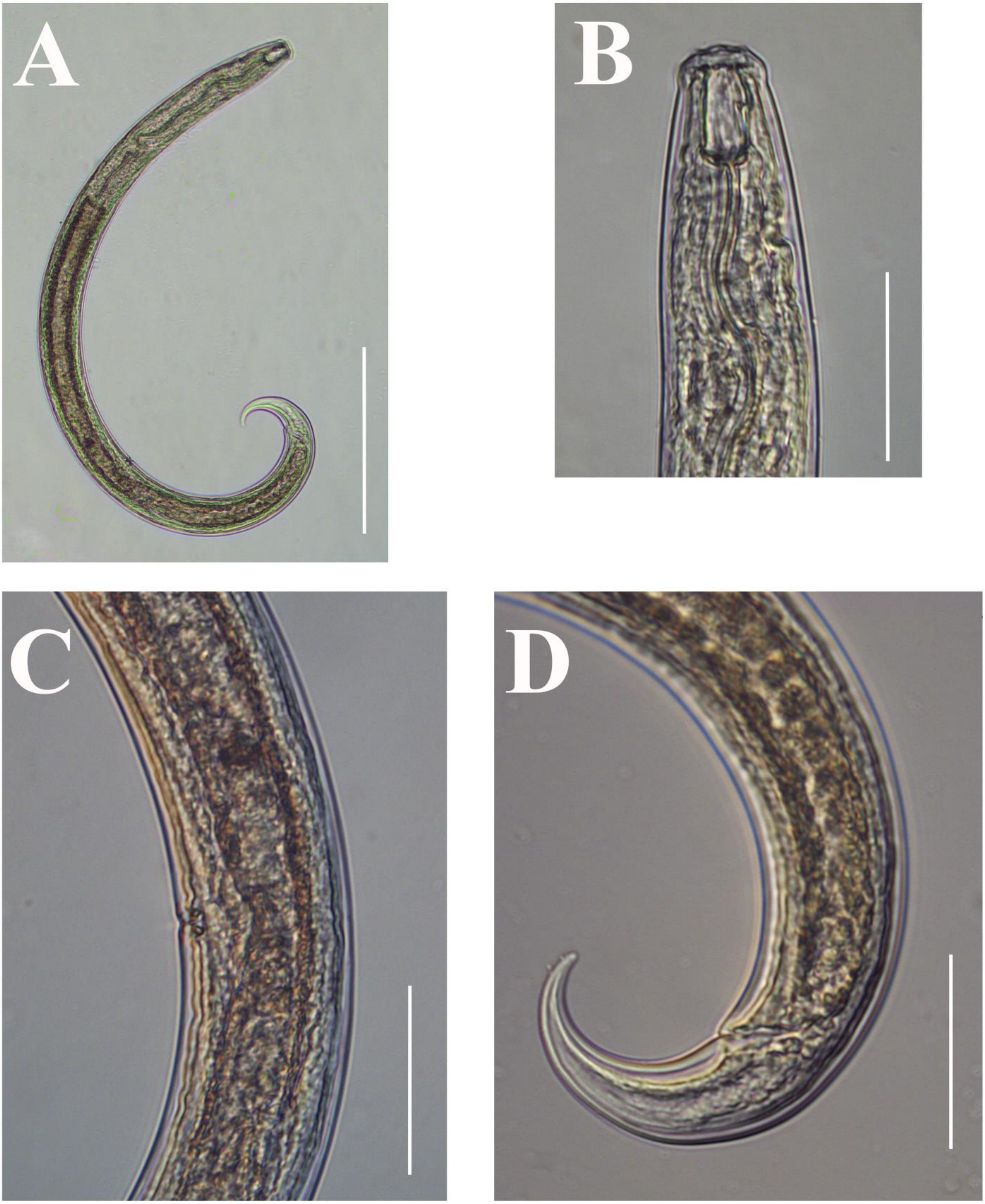

Supplementary Figure S1. Photomicrographs of a sequenced female of *Coomansus parvus* (de Man, 1880) Jairajpuri & Khan, 1977 (SD55), a specimen collected from the riverbank of River Arda. **A** Body, total view. **B** Anterior region. **C** Vulval region showing *pars refringens vaginae*. **D** Tail. *Scale-bars*: **A**, 200 µm; **B-D**, 50 µm.

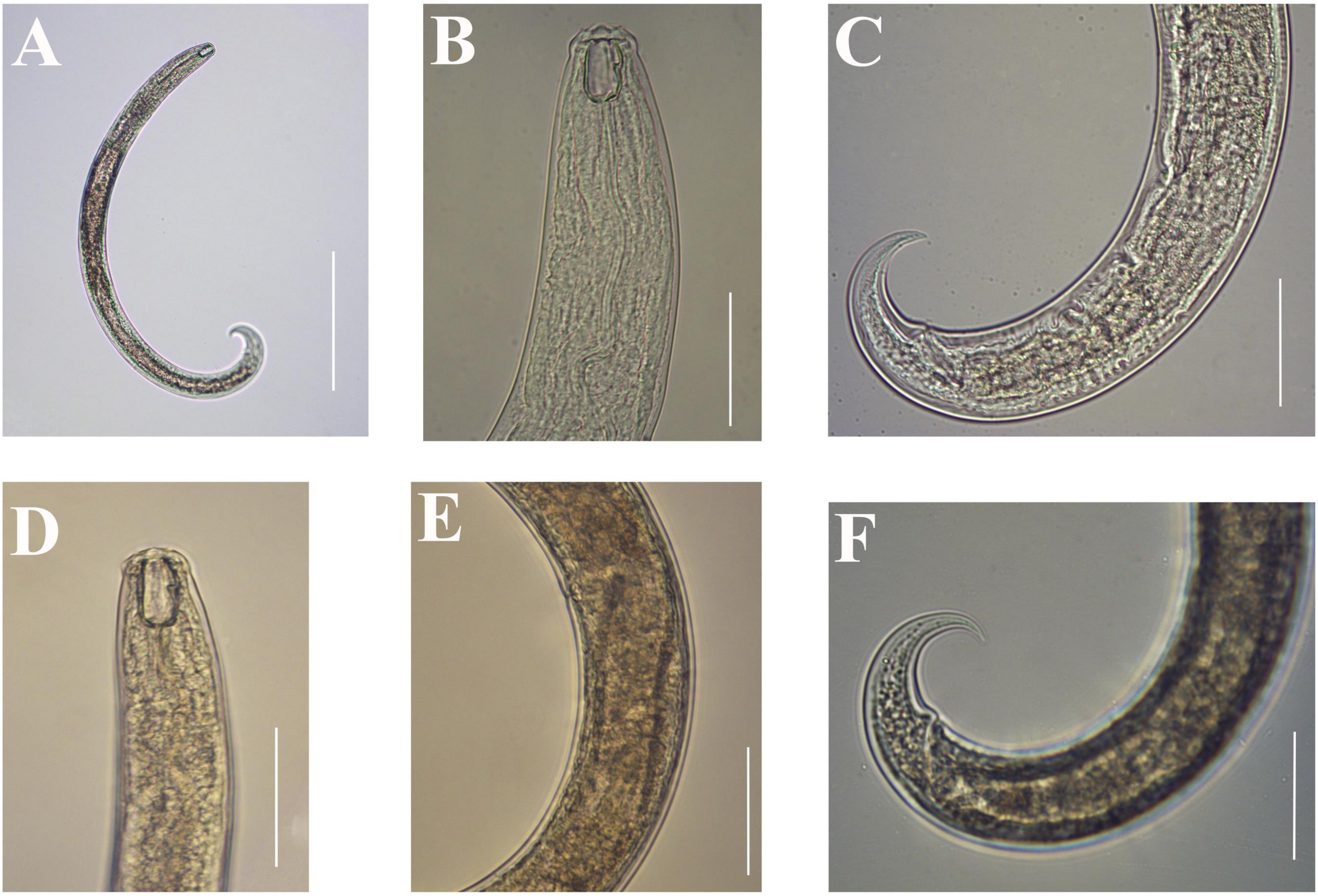

Supplementary Figure S2. Photomicrographs of sequenced females of *Coomansus parvus* (de Man, 1880) Jairajpuri & Khan, 1977 (SD66 and SD67), 2 specimens collected from the riverbank of River Vedena **A-C** SD66, **D-F** SD67; **A** Body, total view. **B-D** Anterior region. **E** Vulval region showing *pars refringens vaginae* and posterior genital branch. **C**, **F** Tail. *Scale-bars*: **A**, 200 µm; **B-F**, 50 µm.

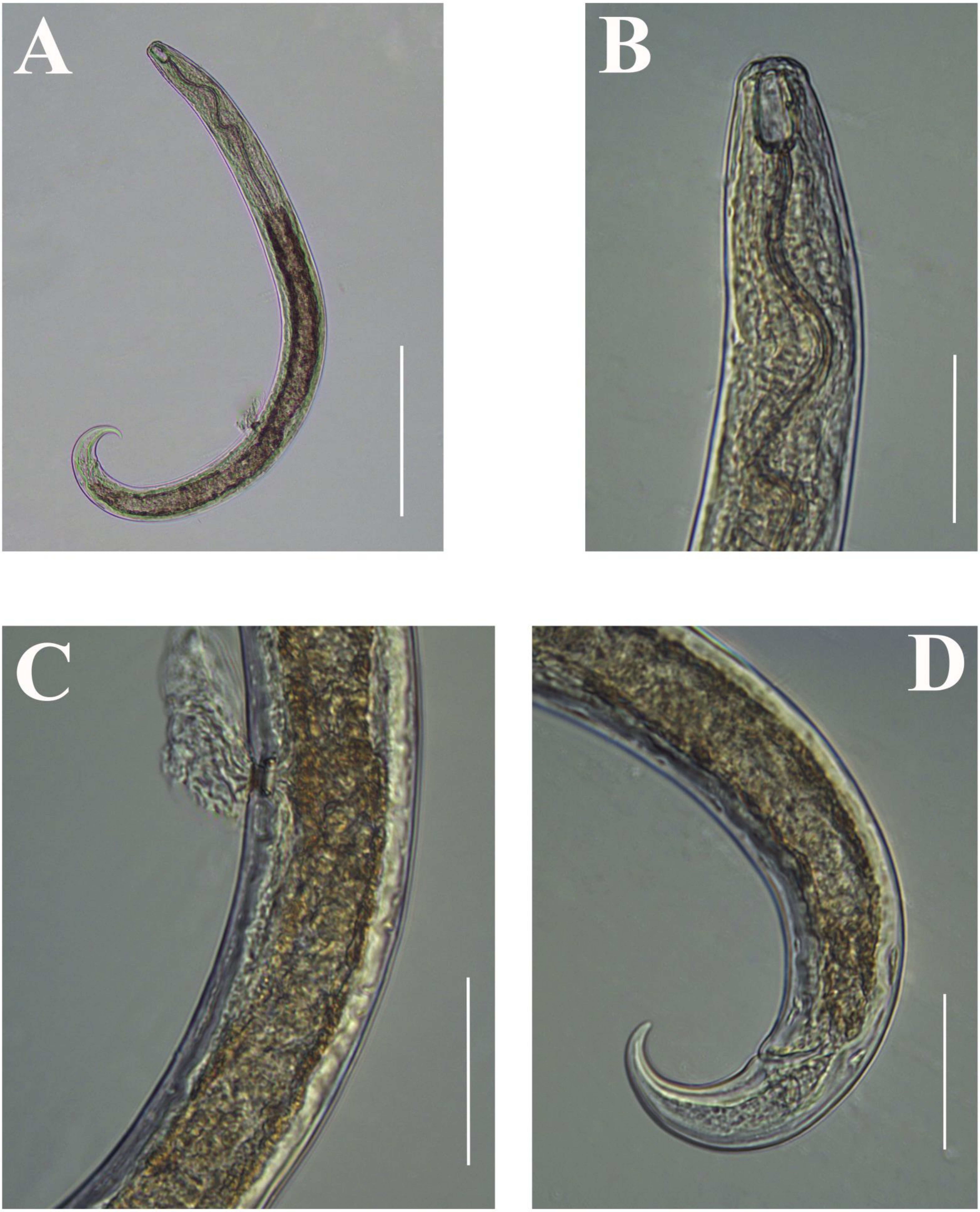

Supplementary Figure S3. Photomicrographs of a sequenced female of *Coomansus parvus* (de Man, 1880) Jairajpuri & Khan, 1977 (SD54M), a specimen collected from the riverbank of River Devinska. **A** Body, total view. **B** Anterior region. **C** Vulval region showing *pars refringens vaginae*. **D** Tail. *Scale-bars*: **A**, 200 µm; **B**-**F**, 50 µm.

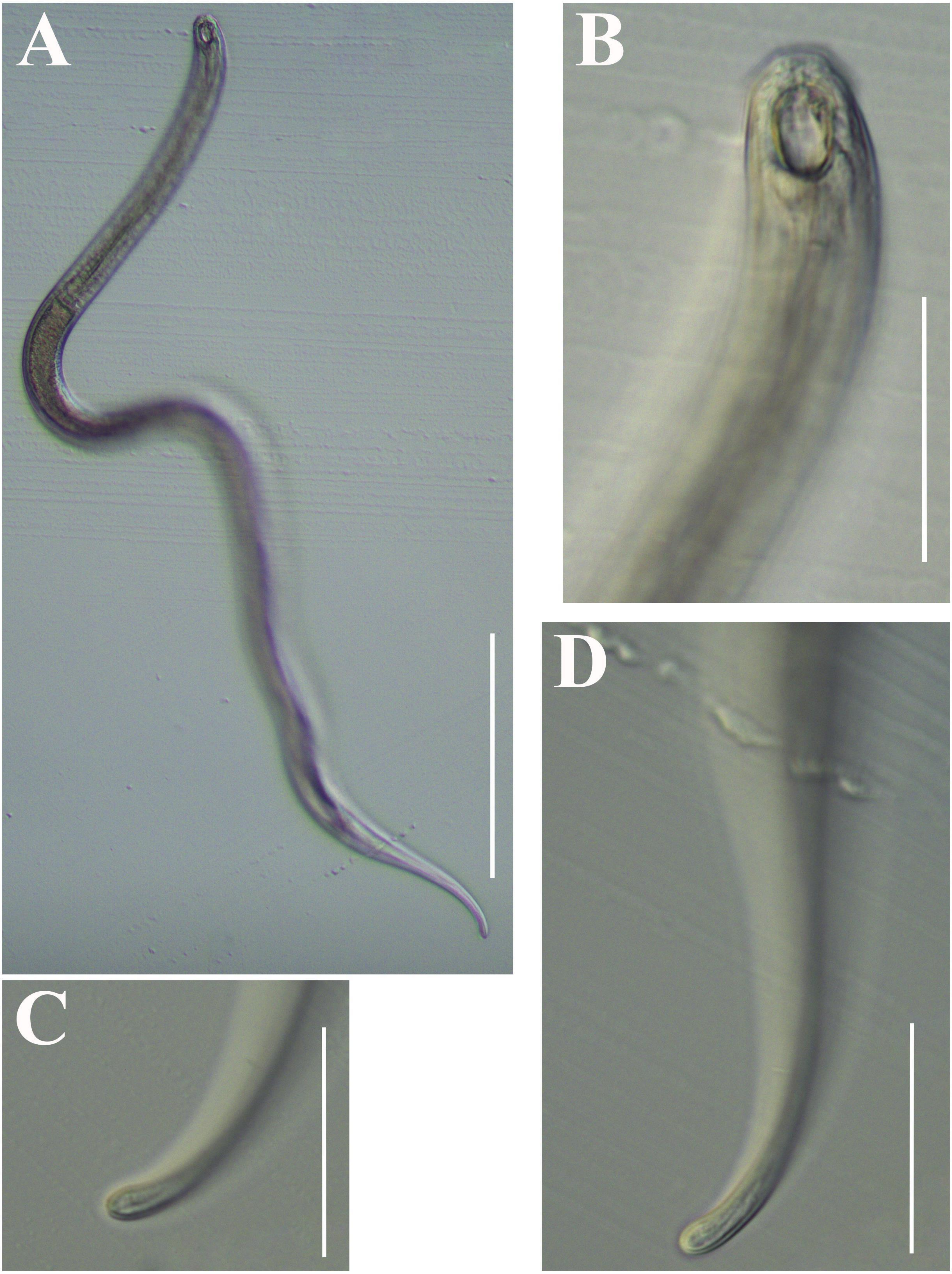

Supplementary Figure S4. Photomicrographs of *Mononchus pseudoaquaticus* sp. nov. from the type-population collected from the riverbank of River Dunabe (SD63). **A** Body, total view. **B** Anterior region. **C** Tail tip. **D** Tail. *Scale-bars*: **A**, 200 µm; **B**-**D**, 50 µm.

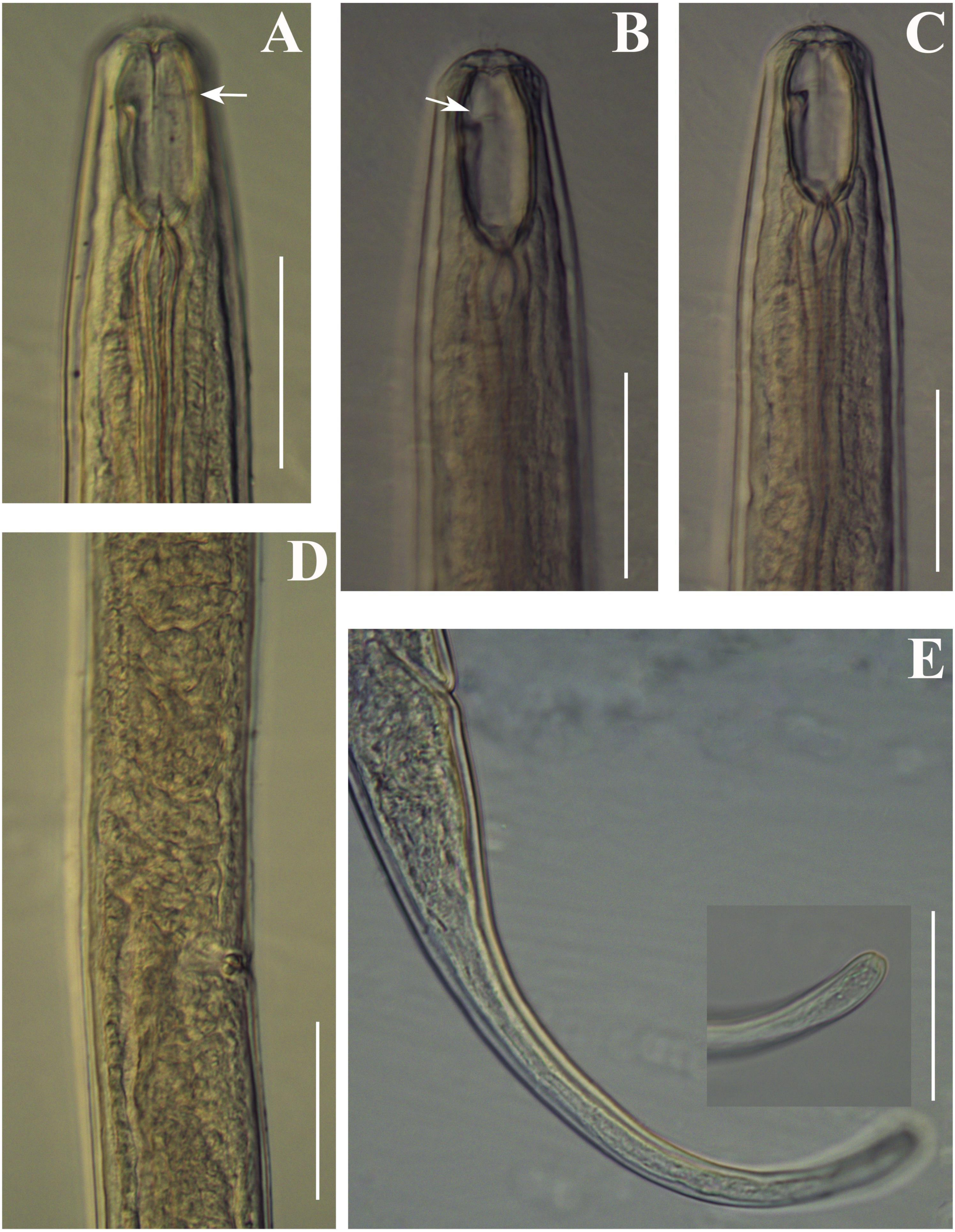

Supplementary Figure S5. Photomicrographs of a sequenced female of *Mononchus truncatus* Bastian, 1865, a specimen collected from the riverbank of River Shirokoleshka (SD43). **A**-**C**, Anterior region (ventro-sublateral ribs arrowed in **A**; amphidial opening arrowed in **B**). **D** Vulval region showing *pars refringens vaginae*. **D** Tail (inset: tail tip). *Scale-bars*: 50 μm.

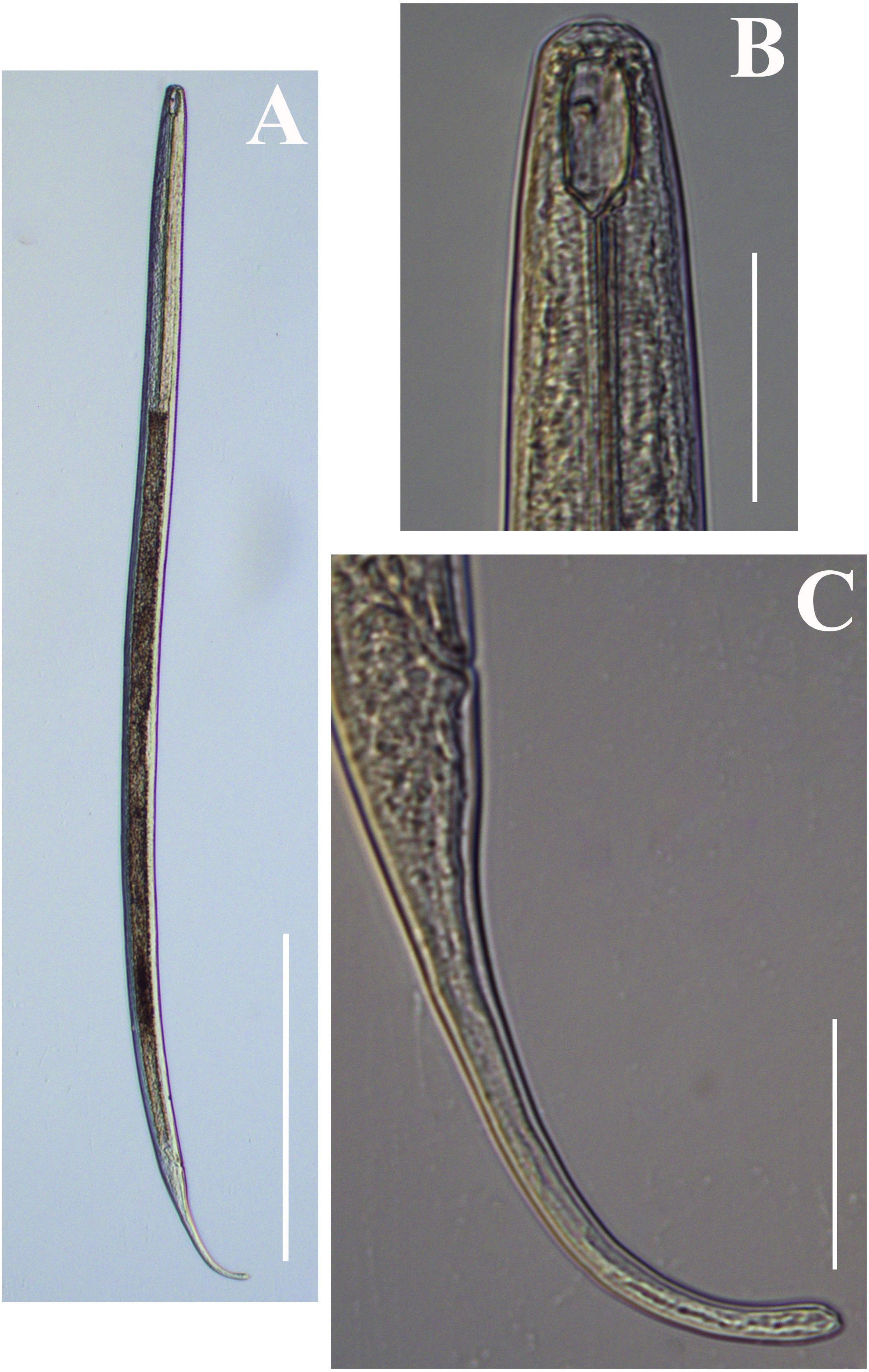

Supplementary Figure S6. Photomicrographs of a sequenced femaile of *Mononchus truncatus* Bastian, 1865, a specimen collected from the riverbank of River Dyavolska (SD64). **A** Body, total view. **B** Anterior region. **C** Tail. *Scale-bars*: **A**, 400 µm; **B**, **C**, 50 µm.

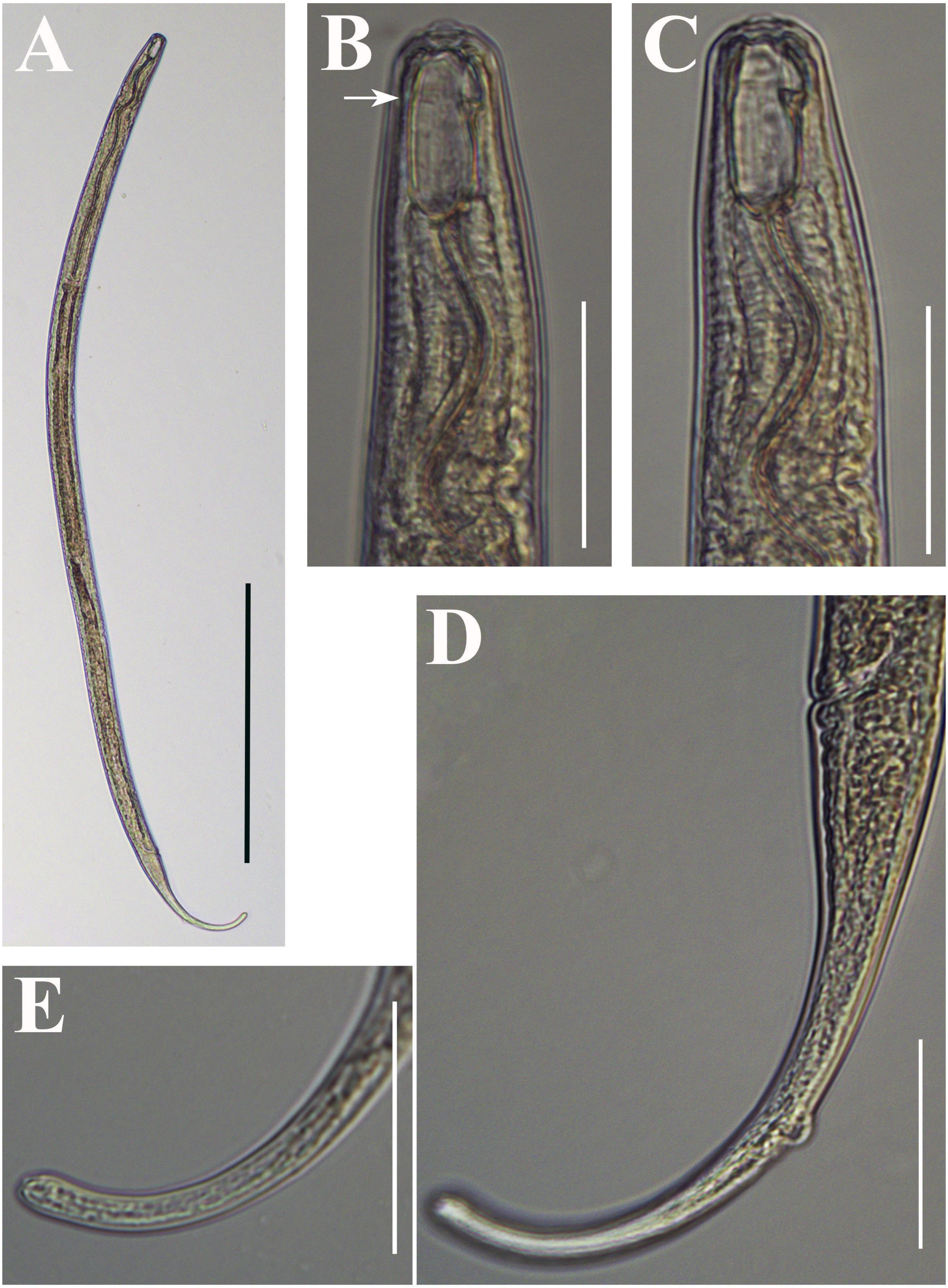

Supplementary Figure S7. Photomicrographs of a sequenced female of *Mononchus truncatus* Bastian, 1865, a specimen collected from the riverbank of River Veleka (SD65). **A** Body, total view. **B**, **C** Anterior region (ventro-sublateral ribs arrowed in **B**). **D** Tail. **E** Tail tip. *Scale-bars*: **A**, 400 µm; **B**-**E**, 50 µm.
