# Supplementary material for: ﻿Integrative taxonomic study of mononchid nematodes from riparian habitats in Bulgaria. I. Genera Mononchus Bastian, 1865 and Coomansus Jairajpuri & Khan, 1977 with the description of Mononchuspseudoaquaticus sp. nov. and a key to the species of Mononchus
Source: Zookeys. 2024 Jul 5;1206:137–80. doi: 10.3897/zookeys.1206.124237 (PMC11245642; doi:10.3897/zookeys.1206.124237)
Supplement: Supplementary material 2 — Comparative morphometric data for females of Coomansusparvus, Mononchusaquaticus, M.pseudoaquaticus sp. nov., and Mononchustruncatus [file zookeys-1206-137_article-124237__-s002.pdf]

Supplementary Table S1 Comparative morphometric data for females of *Coomansus parvus*.

**Source**	**Present study**	**Andrássy (2011b)**	**de Bruin & Heyns (1992)**	**Ahmad & Jairapuri (2010)**	**Iliev & Ilieva (2016)**	**Zullini et al. (2002)**	**Tabolin & Kolganova (2020)**	**Ishaque et al. (2022)**
n	**(*n* = 10)**	**(*n* = na)**	**(*n* = 5)**	**(*n* = na)**	**(*n* = 22)**	**(*n* = 2)**	**(*n* = 15)**	**(*n* = 15)**
L (mm)	0.70–1.15	0.70–1.20	0.90–1.14	0.70–1.40	0.60–1.00	0.81–1.25	0.74–0.94	1.27–1.52
a	12.9–21.7	17.0–26.0	22.0–28.3	17.0–29.0	15.0–23.0	22.0–27.0	16.0–19.0	21.3–25.1
b	3.2–3.7	2.9–4.5	3.3–4.0	2.9–4.5	3.0–3.7	3.5–3.8	3.1–3.7	3.5–3.8
c	11.5–14.8	10.0–19.0	11.5–12.3	10.0–19.0	10.0–17.0	16.0–19.0	12.0–16.0	14.5–18.9
c'	2.0–2.7	1.8–3.4	3.1–3.4	2.0–3.0	1.6–3.6	2.6–3.3	2.1–2.3	2.1–2.8
V (%)	59.6–64.5	58–67	60–67	58–67	62.0–68	64–68	59.5–67.9	67.3–71.1
G1 (%)	9.2–17.3	–	–	–	–	10.0–17.0	9.6–14.8	8.8–11.8[c]
G2 (%)	10.2–15.1	–	–	–	–	9.0–17.0	11.5–13.8	7.8–8.5[c]
Buccal capsule length	22–27	22–26	24–26	21–32	22–27	25–29	24–25	30–34
Buccal capsule width	14–15	10–12	9–13	10–16	13–17	11–12	10–13	26–35 (16–18)[d]
Tooth apex from anterior end of buccal capsule	9–11	–	–	–	–	–	–	10–12
Position of tooth apex (%)[a]	32–38	34–42	27–36	32–41	33–42[b]	36–28[b]	38–44[b]	22–24
Excretory pore from anterior end	97–131	–	–	–	–	–	–	–
Pharynx length	189–324	–	–	–	–	257–327	218–274	–
Lip region height	7–10	–	7–11	–	–	–		–
Lip region width	22–28	20–24	22–23	–	18–23	20–24	20–24	30–34
Amphid from anterior end	10–14	–	8–12	–	14–17	9–12	–	–
Body diameter at pharynx base	43–51	–	–	–	33–55	–	–	50–62
Body diameter at mid-body	47–53	–	–	–	31–57	37–47	–	52–71
Body diameter at anus	28–36	–	–	–	19–30	21–30	–	32–37
Vagina length	12–18	–	–	–	–	–	–	–
Rectum length	21–26	–	18–24	–	16–26	20	–	26–32
Tail length	60–85	50–80	60–93	56–105	45–73	70–78	55–69	80–90

[a] Distance from tooth apex to anterior end of buccal capsule as % of buccal capsule length from its anterior end.
[b] Recalculated from published data.
[c] Values from the description by Ishaque et al. (2022); these differ from the likely erroneous values given in their Table 1 (*G1* = 110–150; *G2* = 112–150).
[d] Numbers from Table 1 of Ishaque et al. (2022) followed by numbers from the description in the same paper in parentheses.

Supplementary Table S2 Comparative morphometric data for females of *Mononchus pseudoaquaticus* sp. nov. and *Mononchus aquaticus*.

**Source**	**Present study**	**Coetzee (1968)**	**Baqri and Jairajpuri (1972)**	**Winiszewska -Ślipińska (1985)**	**Botha & Heyns (1992)**	**de Bruin & Heyns (1992)**	**Coomans et al. (1995)**	**Nakazawa (1999)**	**Eisendle (2008)**	**Farahmand et al. (2009)**	**Zullini et al. (2002)**
n	**(*n* = 10)**	**(*n* = 27)**	**(n = 37)**	**(*n* = 6)**	**(*n* = 23)**	**(*n* = 23)**	**(*n* = 1)**	**(*n* = 13)**	**(*n* = 20)**	**(*n* = 14)**	**(*n* = 1)**
L (mm)	1.23–1.88	1.20–2.00	1.30–1.79	1.00–1.40	1.11–1.45	1.31–2.2	1.37	1.34–1.80	1.10–1.69	1.52–1.94	1.39
a	20.2–33.6	19–35	22–37	23–27	32.6–44.0	31.4–41.1	35.5	28.8–36.5	17.7–30.6	22.9–29.5	31.0
b	4.0–4.7	2.0–5.0	4.2–5.0	3.8–4.5	4.2–5.0	4.3–5.3	4.7	3.6–4.6	3.6–4.8	3.7–5.2	4.5
c	7.2–10.2	8–11	9–12[a]	8–9	9.2–11.8	7.7–10.6	11.7	8.0–9.2	6.8–9.5	7.6–9.8	11.0
c'	4.7–5.8	5.5[c]	–	4–5	4.7–5.6	4.3–6.7	5.0	4.6–6.1	4.1–5.7	4.3–5.9	5.0
V (%)	48.3–53.9	47–55	46–59	51–55	49.6–55.0	48–54	5.0	45.8–57.7	49–54	46.2–55.4	54
G1 (%)	8.0–12.9	7–14	8–19	–	–	–	–		6–13	7–13	14
G2 (%)	7.5–13.3	8–13	6–15	–	–	–	–		11–16	6.3–14.4	15
Buccal capsule length	29–33	30.6[c]	29–31[a]	26–34	24–28	29–36	28	27–34	28–32	25–32	26
Buccal capsule width	15-16	13[c]	14–16[a]	12–16	8–12	11–13	12	13–19	11–14	14–20	9
Tooth apex from anterior end of buccal capsule	5–7	6[c]	–	–	–	–	11	–	–	–	–
Position of tooth apex (%)[b]	18–21	21–25	19–23[a]	25	18–22	16–22	21	20–32	18–25[b]	16–26[c]	23[c]
Excretory pore from anterior end	112–153	–	–	–	–	127–150	–	–	–	–	–
Pharynx length	305–420	–	–	230–284	260–310	–	291	–	–	308–420	307
Lip region height	7–10	10[c]	6–8	–	–	–	9	–	–	7–11	–
Lip region width	23–26	25[c]	21–22	–	17–21	–	20	–	21–27	22–29	19
Amphid from anterior end	8–12		8–11[a]	–	–	7–13	10	–	11	7–10	–
Body diameter at pharynx base	43–62	–		–	–	–	–	–	44–68	–	–
Body diameter at mid-body	44–71	–		–	30–39	–	–	42–60	47–78	53–77	45
Body diameter at anus	30–40	33[c]		–	23–27	–	–	–	27–43	35–42	25
Vagina length	15–20	20[c]		–	–	–	–	–		12–21	–
Rectum length	26–31	35[c]	25–32	–	19–28	18–30	–	–	19–38	32–39	22
Tail length	171–210	180[c]	94–156	117–178	106–143	140–240	117	167–216	152–208	168–217	126

[a] Including data from a re-examination of paratypes of *M. aquaticus*
[b] Distance from tooth apex to anterior end of buccal capsule as % of buccal capsule length from its anterior end.
[c] Calculated from published data.

Supplementary Table S3 Comparative morphometric data for females of *Mononchus pseudoaquaticus* sp. nov. and morphologically similar species.

**Species**	***M. pseudoaquaticus***	***M. aquaticus* (*sensu stricto*)**			***M. caudatus***	***M. pulcher***	
**Source**	**Present study**	**Coetzee (1968)**	**Baqri & Jairajpuri (1972)**	**Andrássy (2011a)**	**Shah & Hussain (2016)**	**Andrássy (1993)**	
n	**(*n* = 10)**	**(*n* = 27)**	**(*n* = 37)**	**–**	**(*n* = 6)**	**Chile (*n* = 7)**	**Hungary (*n* = na)**
L (mm)	1.23–1.88	1.20–2.00	1.30–1.79	1.20–1.70	1.73–1.92	1.72–2.04	1.94–2.10
a	20.2–33.6	19–35	22–37	20–37	34–38	35–39	35–38
b	4.0–4.7	2–5	4.2–5.0	4.2–5.0	4.0–5.0	4.5–4.8	4.4–4.9
c	7.2–10.2	8–11	9–12[a]	8–12	9–10	9.2–9.7	8.7–9.4
c'	4.7–5.8	5.5[b]	–	5–6	5–6	6.0–6.5	6–7
V (%)	48.3–53.9	47–55	46–59	46–59	48–51	50–52	49–51
G1 (%)	8.0–12.9	7–14	8–19	–	–	–	–
G2 (%)	7.5–13.3	8–13	6–15	–	–	–	–
Buccal capsule length	29–33	30.6[c]	29–31[a]	26–32	30–33	35–38	32–33
Buccal capsule width	15–16	13[c]	14–16[a]	–	15–17	16–18	16–17
Tooth apex from anterior end of buccal capsule	5–7	6[c]	–	–	7 (holotype)	–	–
Position of tooth apex (%)[d]	18–21	21–25	19–23[a]	20–25	18–21	20–22	20–22
Excretory pore from anterior end	112–153	–	–	–	132–142	–	–
Pharynx length	305–420	–	–	–	386–391	384–410[e]	384–410[e]
Lip region height	7–10	10[c]	6–8	–	5–8	–	–
Lip region width	23–26	25[c]	21–22	18–24	24–25	23–25[e]	23–25[e]
Amphid from anterior end	8–12	–	8–11[a]	–	9–12	–	–
Body diameter at pharynx base	43–62	–	–	–	–	–	–
Body diameter at mid-body	44–71	–	–	–	45–53	51–56[e]	51–56[e]
Body diameter at anus	30–40	33[c]	–	–	–	–	–
Vagina length	15–20	20[c]	–	–	27–29	–	–
Rectum length	26–31	35[c]	25–32	–	32–36	–	–
Tail length	171–210	–	94–156	120–200 (mean 150)	190–195	186–225[e]	186–225[e]

[a] Including data from a re-examination of paratypes of *M. aquaticus*
[b] Calculated from published data.
[c] Measured from the original drawing.
[d] Distance from tooth apex to anterior end of buccal capsule as % of buccal capsule length from its anterior end.
[e] Data in the original description of Andrássy (1993) cannot be separated for the Chilean and Hungarian populations.

Supplementary Table S4 Comparative morphometric data for females of *Mononchus truncatus* (*sensu lato*).

**Source**	**Present study**	**Clark (1960)[a]**	**Andrássy, (2011a)[b]**	**Botha and Heyns (1992)**	**de Bruin & Heyns (1992)**	**Coomans et al. (1995)**	**Nakazawa (1999)**	**Zullini et al. (2002)**	**Eisendle (2008)**	**Farahmand et al. (2009)**	**Ahmad & Jairapuri (2010)**
n	**(*n* = 14)**	**(*n* = 12)**	**(*n* = 22)**	**(*n* = 8)**	**(*n* = 6)**	**(*n* = 1)**	**(*n* = 131)**	**(*n* = 10)**	**(*n* = 20)**	**(*n* = 9)**	**(*n* = na)**
L	1.77–2.09	1.65–2.14	1.72–2.12	1.66–2.04	1.66–2.10	1.94	1.42–2.45	1.61–2.01	1.41–2.14	1.72–2.44	1.65–2.40
a	26.5–34.0	26–40	27–35	30.9–36.9	31.3–40.1	30.4	25.0–41.7	27–33	19.9–32.7	24.6–34.9	26–40
b	3.7–4.3	3.4–4.3	3.8–4.3	3.5–4.6	3.6–4.1	4.1	3.3–4.7	3.8–4.2	3.3–4.2	4.0–4.7	3.3–4.3
c	7.8–9.3	5.8–8.6	7.5–8.4	7.4–10.6	7.1–8.6	8.3	5.5–11.3	7.3–11.0	7.2–11.1	7.3–10.3	5.5–8.6
c'	5.0–6.7	6–8	6.3–7.6	5.3–7.6	6.6–8.0	7.0	4.5–11.5	4.6–7.8	3.5–6.2	5.1–7.2	6–8
V (%)	52.6–57.6	48.4–55.5	52–56	52.4–59.5	52–55	54	47.2–61.7	50–55	52–57	49.7–54.9	48–56
G1 (%)	9.5–11.3	9–22	–	–	–	–	–	7–12	6–13	7.5–13	–
G2 (%)	9.7–11.8	7–15	–	–	–	–	–	9–13	11–16	10–13	–
Buccal capsule length	40–44	43–50	42–46	38–48	42–49	46	38–51	39–43	38–48	32–46	40–50
Buccal capsule width	18–22	19[c]	18–21	15–18	14–15	20	14–21	13–15	15–19	15–21	18–22
Tooth apex from anterior end of buccal capsule	10–12	12[c]	10–12	–	–	–	–	–	–	–	–
Position of tooth apex (%)[d]	25–29	23–28	25–29	17–30	21–29	31	15–33	24–30[b]	25–36[c]	17–27[c]	30–33[b]
Excretory pore from anterior end	134–176	–	–	–	–	154	–	–	–	–	–
Pharynx length	423–525	–	444–506	410–550	–	465	–	415–490	–	399–564	–
Lip region height	8–11	10[c]	–	–	–	10	–	–	–	7–11	6–8
Lip region width	25–30	27–28	24–25	25–30	23–28	29	–	23–26	25–30	25–31	22–29
Amphid from anterior end	10–13	–	–	–	8–10	10	–	–	10	6–11	–
Body diameter at pharynx base	51–61	–	60–62	–	–	–	–	–	51–81	–	–
Body diameter at mid-body	53–71	–	62–74	49–65	–	–	42–76	49–70	55–95	60–88	–
Body diameter at anus	34–47	47[c]	31–36	33–38	–	–	–	27–40	35–52	35–46	–
Vagina length	14–18	–	–	–	–	–	–	–		23–25	–
Rectum length	27–36	35[c]	–	29–38	29–33	–	–	28–37	32–42	35–39	–
Tail length	205–254	240–283	232–280	179–272	204–250	232	171–335	185–234	136–234	196–280	250–280

[a] Neotype population, including the measurements of the neotype by Coomans and Khan (1981) and of the neotype populations by Baqri and Jairajpuri (1972).

[b] Population of the "real" *M. truncatus sensu* Andrássy (2011a) from the littoral zone of Lake Balaton, Hungary.

[c] Calculated from published description/drawings.

[d] Distance from tooth apex to anterior end of buccal capsule as % of buccal capsule length from its anterior end.

Supplementary Table S5 Comparative morphometric data for females of *Mononchus truncatus* (*sensu stricto*).

**Source**<br>***n***	**Present study**<br>**(*n* = 14)**	**Clark (1960)[a]**<br>**(*n* = 12)**	**Andrássy, (2011a)[b]**<br>**(*n* = 22)**
L	1.77–2.09	1.65–2.14	1.72–2.12
a	26.5–38.9	26–40	27–35
b	3.7–4.3	3.4–4.3	3.8–4.3
c	7.8–9.3	5.8–8.6	7.5–8.4
c'	5.0–6.7	6.0–8.0	6.3–7.6
V (%)	52.6–57.6	48.4–55.5	52–56
G1 (%)	9.5–11.3	9–22	–
G2 (%)	9.7–11.8	7–15	–
Buccal capsule length	40–44	43–50	42–46
Buccal capsule width	18–22	19[c]	18–21
Tooth apex from anterior end of buccal capsule	10–12	12[c]	10–12
Position of tooth apex (%)[d]	25–29	23–28	25–29
Excretory pore from anterior end	134–176	–	–
Pharynx length	423–525	–	444–506
Lip region height	8–11	10[c]	–
Lip region width	25–30	27–28	24–25
Amphid from anterior end	10–13	–	–
Body diameter at pharynx base	51–61	–	60–62
Body diameter at mid-body	53–71	–	62–74
Body diameter at anus	34–47	47[c]	31–36
Vagina length	14–18	–	–
Rectum length	27–36	35[c]	–
Tail length	205–254	240–283	232–280

[a] Neotype population, including the measurements of the neotype by Coomans and Khan (1981) and of the neotype populations by Baqri and Jairajpuri (1972).
[b] Population of the “real” *M. truncatus sensu* Andrássy (2011a) from the littoral zone of Lake Balaton, Hungary.
[c] Calculated from published description/drawings.
[d] Distance from tooth apex to anterior end of buccal capsule as % of buccal capsule length from its anterior end.

References

Ahmad W, Jairajpuri MS (2010) Mononchida: The predaceous nematodes. In: Hunt DJ, Perry RN (Eds), Nematology Monographs and Perspectives; v. 7. Brill Academic Publishers, Leiden-Boston, 298 pp.Andrássy (1993)S3

Andrássy I (1993) A taxonomic survey of the family Mononchidae (Nematoda). Acta Zoologica Hungarica 39: 13–60.

Andrássy I (2011a) Three new species of the genus *Mononchus* (Nematoda: Mononchida), and the “real” *Mononchus truncatus* Bastian. Journal of Natural History 45: 303-326. https://doi.org/10.1080/00222933.2010.524947

Andrássy I (2011b) Three new species of the genus *Coomansus* Jairajpuri & Khan, 1977 from the southern hemisphere (Nematoda: Mononchida). Journal of Nematode Morphology and Systematics 14: 27–37.

Baqri SZ, Jairajpuri MS (1972). Studies on Mononchida of India V. Some observations on the morphology of *Mononchus aquaticus* Coetzee, 1968 with remarks on its status. Indian Journal of Nematology 2: 105–116.

Botha A, Heyns J (1992) Further records and descriptions of nematodes from rivers in the Kruger National Park (Orders Enoplida, Chromadorida, Monhysterida, Mononchida and Araeolaimida). Koedoe 35: 11–25. https://doi.org/10.4102/koedoe.v35i2.401

Clark WC (1960) Redescription of *Mononchus truncatus* Bastian, *M. papillatus* Bastian and *Prionchulus muscorum* (Dujardin) (Enoplida, Nematoda). Nematologica 5: 184–198.

Coetzee V (1968) Southern African species of the genera *Mononchus* and *Prionchulus* (Mononchidae). Nematologica 14: 63–76.

Coomans A, Rashid F, Heyns J (1995) On some predatory nematodes from the Okavango Delta, Botswana. Hydrobiologia 302: 119–131. https://doi.org/10.1007/BF00027037

De Bruin S, Heyns J (1992). Mononchida (Nematoda) of Southern Africa: Genera *Mononchus* Bastian, 1865, *Clarkus*, Jairajpuri, 1970 and *Coomansus* Jairajpuri & Khan, 1977. Phytophylactica 24: 61–73. https://journals.co.za/doi/pdf/10.10520/AJA03701263_1459

Eisendle U (2008) Description of *Mononchus sandur* n. sp. (Nematoda: Mononchidae) and remarks on *M. truncatus* Bastian, 1865 and *M. aquaticus* Coetzee, 1968 from a glacial floodplain reach (Großglockner region, Hohe Tauern, Austria). Nematology 10: 809–818.

Farahmand S, Eskandari A, Orselli L, Karegar A. 2009. Some known species of the genera *Mononchus* Bastian, 1865 and *Mylonchulus* (Cobb, 1916) Altherr, 1953 (Nematoda: Mononchina) from Semnan province, Iran. Nematologia Mediterranea 37: 145–154.

Iliev I, Ilieva Z (2016) New data on species of order Mononchida (Nematoda) from Rila and the Rhodopes Mountains, Bulgaria. Silva Balcanica 17: 63–84.

Ishaque U, Iqbal E, Dawar S, Kazi N (2022) Description of *Mononchus oryzae* n. sp. with observation on *Coomansus parvus* (De Man, 1880) Jairajpuri and Khan, 1977 (Mononchida: Mononchidae) from Pakistan. Pakistan Journal of Zoology 2022: 1–4.

Koohkan M, Shokoohi E, Abolafia J (2014) Study of some mononchids (Mononchida) from Iran with a compendium of the genus *Anatonchus*, Tropical Zoology 27: 88–127. DOI: 10.1080/03946975.2014.966457

Nakazawa K (1999) Morphological characteristics and ecological survey of three species of the genus *Mononchus* from wet soil in Gunma Prefecture. Japanese Journal of Nematology 29:16–23.

Shah AA, Hussain A (2016) Descriptions of three new species of *Mononchus* (Nematoda: Mononchida) from Jammu and Kashmir State, India. International Journal of Nematology 26: 29–40.

Tabolin SB, Kolganova TV (2020) Characterisation of *Coomansus parvus* (de Man, 1880) from Russia with the first report of males. Russian Journal of Nematology 28: 131–134.

Winiszewska-Ślipińska G (1985) Sześć gatunków Mononchoidea (Nematoda) nowych dla fauny Polski. Państwowe Wydawnictwo Naukowe 29: 29–38. http://rcin.org.pl/miiz/dlibra/publication/edition/52093

Zullini A, Loof PAA, Bongers T (2002) Free-living nematodes from nature reserves in Costa Rica 2. Mononchina. Nematology 4: 1–23.
